# Carbon dot/TiO_2_ nanocomposites for photocatalytic antibiotic degradation: interfacial engineering, degradation mechanisms, and environmental remediation

**DOI:** 10.1039/d6ra08578d

**Published:** 2026-10-28

**Authors:** Sewara J. Mohammed, Eman O. Hamarashid, Hastyar H. Najmuldeen, Dana A. Kader, Mohammed K. Sidiq, Faiq H. S. Hussain, Awat S. Mohammed, Fayez Alghofaili, Govar H. Hamasalih, Shakhawan A. Mhamad, Yar M. Faqi-Khedr, Kawan F. Kayani, Othman K. Hamaamin, Shujahadeen B. Aziz

**Affiliations:** a Department of Chemistry, College of Science, University of Sulaimani Qlyasan Street Sulaymaniyah 46001 Kurdistan Regional Government Iraq sewara.mohammed@univsul.edu.iq; b Department of Medical Laboratory Analysis, College of Health Sciences, Cihan University Sulaimaniya Sulaymaniyah City Kurdistan Iraq; c Department of Biology, College of Science, University of Sulaimani Qlyasan Street Sulaymaniyah 46001 Kurdistan Regional Government Iraq; d Department of Chemistry, College of Education, University of Sulaimani Old Campus Sulaymaniyah 46001 Kurdistan Regional Government Iraq; e Department of Pharmacognosy and Pharmaceutical Chemistry, College of Pharmacy, University of Sulaimani Sulaymaniyah Kurdistan Regional Government 46001 Iraq; f Medical Analysis Department, Faculty of Applied Science, Tishk International University Erbil Kurdistan Region 44001 Iraq; g Department of Medical Laboratory Sciences, College of Applied Medical Sciences, Majmaah University Majmaah 11952 Saudi Arabia; h Department of Physics, College of Education, University of Sulaimani, Kurdistan Regional Government Old Campus Sulaymaniyah 46001 Iraq; i Turning Trash to Treasure Laboratory (TTTL), Research and Development Center, University of Sulaimani Qlyasan Street Sulaymaniyah Kurdistan Regional Government 46001 Iraq

## Abstract

The widespread presence of antibiotic residues in aquatic environments contributes to ecological toxicity and accelerates antimicrobial resistance (AMR), highlighting the need for efficient and sustainable remediation technologies. Among advanced oxidation processes, TiO_2_-based photocatalysis has attracted considerable attention because of its chemical stability, low toxicity, and strong oxidative capability. However, the practical application of TiO_2_ is limited by poor visible-light absorption and rapid electron–hole recombination. Carbon dots (CDs) have emerged as highly promising interfacial modifiers owing to their tunable electronic structure, visible-light responsiveness, and efficient charge-transfer capability, enabling substantial enhancement of TiO_2_ photocatalytic performance. This review critically examines recent advances in CDs/TiO_2_ nanocomposites for photocatalytic antibiotic degradation, with particular emphasis on how heterointerface engineering governs charge-carrier dynamics, reactive oxygen species (ROS) generation, and degradation selectivity. The mechanistic roles of Ti–O–C interfacial bonding, band alignment, defect-state modulation, and interfacial electron transfer are systematically discussed in relation to photocatalytic activity across major antibiotic classes, including tetracyclines, fluoroquinolones, β-lactams, and sulfonamides. In addition, the effects of adsorption behavior, operational parameters, environmental matrix components, and reactor configuration on degradation efficiency and mineralization are critically evaluated. Special attention is devoted to degradation pathways, catalyst photostability, ecotoxicological considerations, and challenges associated with real-water applications and large-scale implementation. Current limitations, including insufficient mechanistic validation, lack of standardized benchmarking protocols, and limited long-term environmental safety assessment, are also highlighted. Finally, current research priorities involving mechanistic validation, predictive interfacial engineering, standardized benchmarking, environmental safety assessment, and solar-driven photocatalytic design are highlighted to guide the future development of sustainable antibiotic-remediation technologies.

## Abstract 2

Effective antibiotic remediation requires linking carbon dot–TiO_2_ interfacial design with charge-transfer mechanisms, degradation pathways, and environmental safety. Image created with assistance from FigureLabs AI.

## Graphical abstract

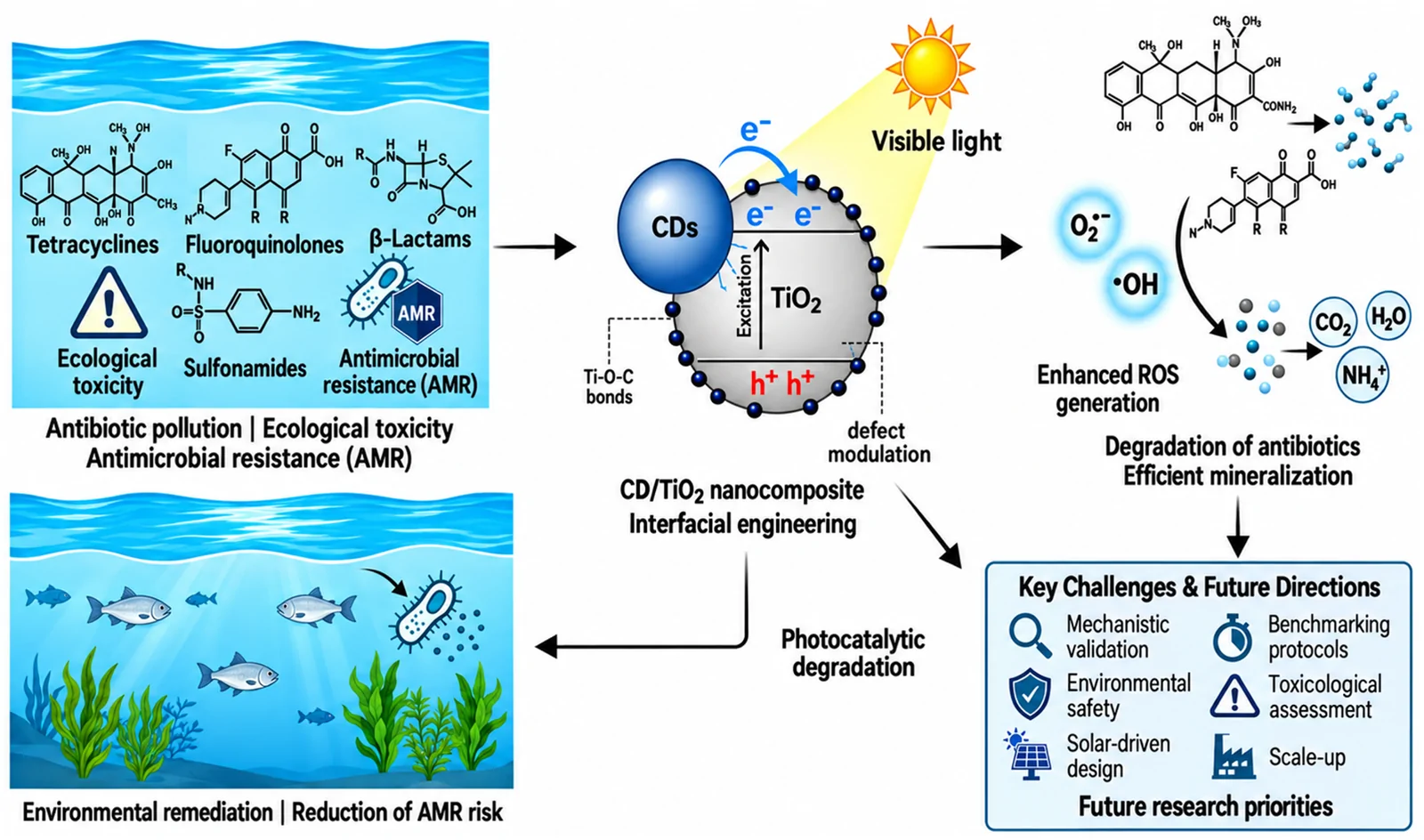

## 1 Introduction

The extensive use of antibiotics in human medicine, veterinary practice, and agriculture has resulted in their persistent detection in aquatic environments.^1–3^ A substantial fraction of administered antibiotics is excreted unmetabolized and enters wastewater streams through hospital effluents, pharmaceutical discharge, livestock runoff, and aquaculture activities.^4–6^ As illustrated in Fig. 1, these sources contribute continuously to environmental antibiotic contamination and accelerate the dissemination of antimicrobial resistance (AMR). Many antibiotics exhibit high environmental persistence and incomplete biodegradability under conventional wastewater-treatment conditions, limiting the effectiveness of traditional biological treatment systems.^7,8^

**Fig. 1:**
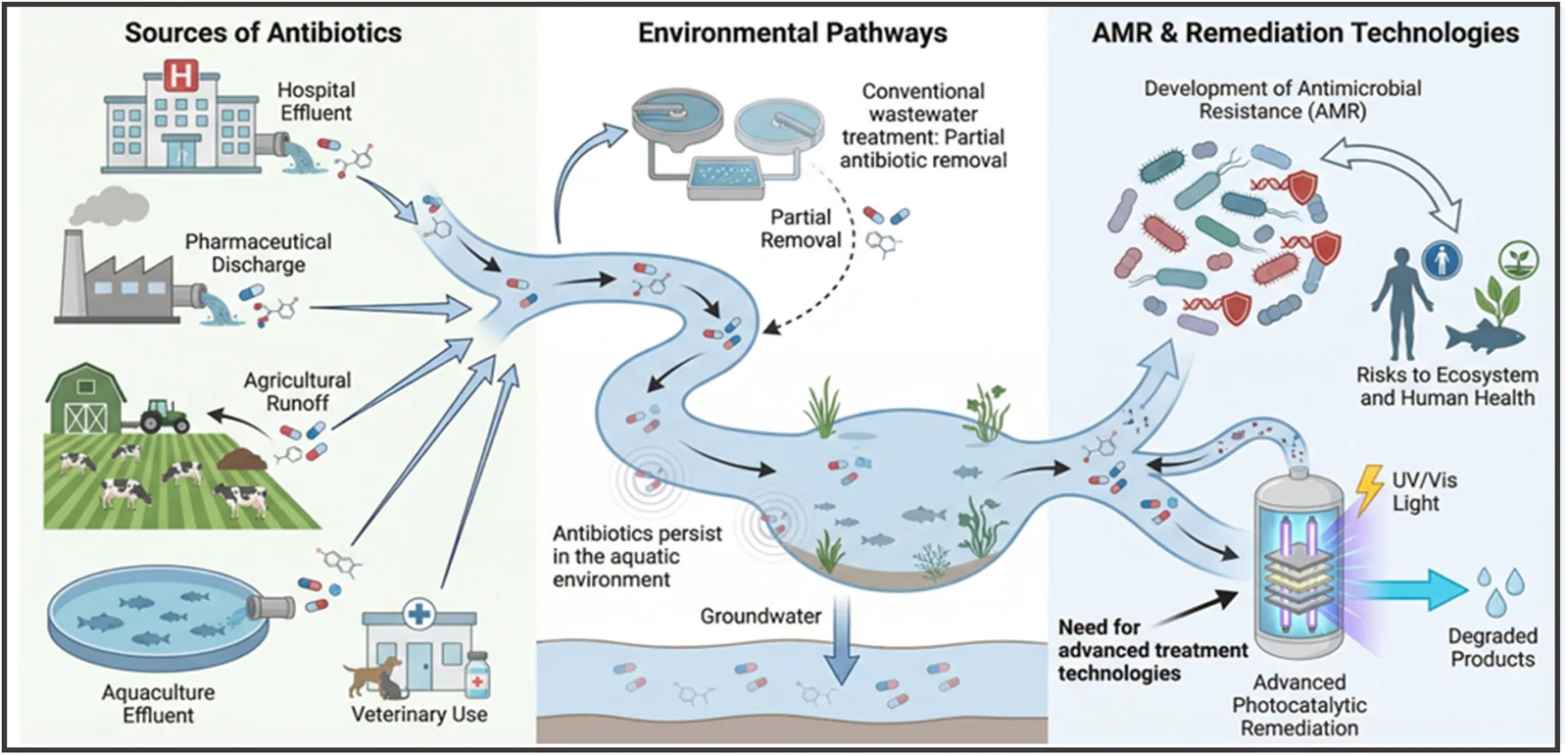
Schematic illustration of major antibiotic emission sources, environmental transport pathways, and the development of antimicrobial resistance (AMR) in aquatic systems. The figure highlights hospital effluents, pharmaceutical discharge, agricultural runoff, and the resulting need for advanced photocatalytic remediation technologies.

The environmental persistence of antibiotics exerts continuous selective pressure on microbial communities, promoting the emergence and propagation of AMR.^9,10^ This growing threat compromises the effectiveness of existing therapeutic agents and has become a major global public-health concern.^11^ Consequently, the development of advanced treatment technologies capable of degrading antibiotics at the molecular level has become an urgent research priority.^12,13^

Among advanced oxidation processes (AOPs), semiconductor photocatalysis has attracted considerable attention because of its ability to generate reactive oxygen species (ROS) under light irradiation.^14–16^ Upon photoexcitation, photogenerated electrons and holes participate in interfacial redox reactions that produce highly oxidative species, including hydroxyl radicals (˙OH), superoxide radicals (O_2_˙^−^), and singlet oxygen (^1^O_2_).^17,18^ These ROS possess strong oxidation potential and can effectively degrade complex aromatic and heterocyclic structures commonly present in antibiotic molecules.^19,20^

Titanium dioxide (TiO_2_) remains one of the most extensively investigated photocatalysts for environmental remediation because of its chemical stability, low toxicity, abundance, and strong oxidative capability.^21,22^ However, the practical application of pristine TiO_2_ is limited by rapid electron–hole recombination and weak visible-light absorption arising from its wide bandgap.^23,24^ These intrinsic limitations have motivated extensive efforts toward TiO_2_ modification through elemental doping, heterojunction construction, and interfacial engineering strategies.^25–28^

Carbonaceous materials such as carbon nanotubes (CNTs), graphene oxide (GO), and carbon dots (CDs) have been widely investigated for TiO_2_ modification because of their ability to promote charge separation and improve photocatalytic performance. CNTs provide efficient charge-transport pathways; however, their strong inter-tube interactions can cause aggregation and poor dispersion in solvents, often requiring surface functionalization to obtain homogeneous composites.^29^ RGO and graphene-based materials can facilitate electron transport and pollutant adsorption. However, restacking of two-dimensional sheets and agglomeration in TiO_2_ composites may reduce the accessible surface area and affect photocatalytic performance.^30^ In comparison, CDs offer several attractive features for TiO_2_-based photocatalysis, including ultrasmall dimensions, high aqueous solubility, facile synthesis and functionalization, broad light absorption, and electron-transfer capability. These characteristics facilitate their integration with TiO_2_ and provide multiple pathways for enhancing light utilization, interfacial charge transfer, and pollutant degradation, which motivates the present focus on CD/TiO_2_ nanocomposites.^31,32^ In this review, the term CDs is used as a general designation for nanoscale carbon-based materials that include carbon quantum dots (CQDs), which are quasi-spherical nanoparticles with amorphous or partially crystalline carbon cores and abundant surface functional groups; graphene quantum dots (GQDs), which consist of one or a few graphene layers with lateral dimensions typically below 20 nm and possess a high surface area, an sp^2^-hybridized lattice, and a planar structure that enable π–π interactions; and polymer-derived CDs, which are synthesized from polymeric precursors and often retain partial polymeric characteristics while exhibiting a high heteroatom content.^33^

Given these characteristics, carbon dots (CDs) have emerged as promising nanomaterials for improving TiO_2_ photocatalysis.^34,35^ As illustrated in Fig. 2, coupling CDs with TiO_2_ creates electronically integrated heterointerfaces that enhance solar-light harvesting, facilitate charge separation, and regulate ROS generation. The tunable surface states, excellent electron-transfer capability, and versatile surface functionalization of CDs make them attractive interfacial modifiers for visible-light-responsive photocatalytic systems.^36–39^

**Fig. 2:**
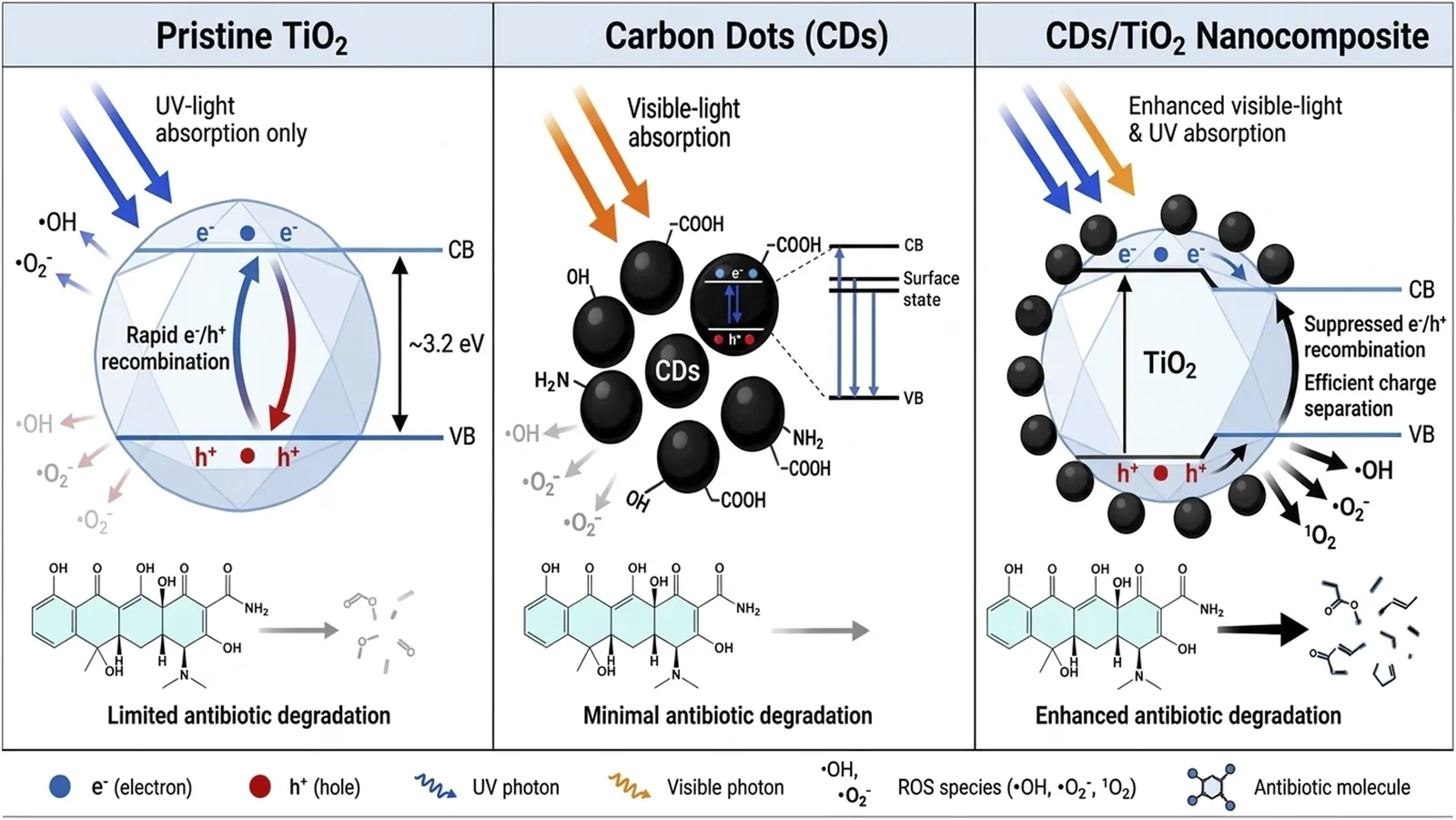
Schematic comparison of pristine TiO_2_, CDs, and CDs/TiO_2_ nanocomposites, highlighting differences in band structure, photon absorption, charge-carrier dynamics, ROS generation, and photocatalytic antibiotic degradation behavior under irradiation conditions.

Numerous studies have reported enhanced photocatalytic performance in CDs/TiO_2_ systems.^32^ The relationship between interfacial electronic structure, ROS generation, and antibiotic-specific degradation behavior remains insufficiently understood. Many previous reviews primarily discuss TiO_2_ photocatalysis or carbon-based nanomaterials independently, while limited attention has been devoted to the mechanistic role of heterointerface engineering in antibiotic degradation.^40–42^ Furthermore, critical challenges, including degradation selectivity, catalyst stability, real-water applicability, and environmental safety, are often addressed separately rather than within an integrated environmental remediation framework.

Therefore, this review provides a critical and mechanistic overview of CDs/TiO_2_ nanocomposites for photocatalytic antibiotic degradation, spanning fundamental material properties, interfacial charge-transfer mechanisms, degradation pathways, and environmental remediation applications. Particular emphasis is placed on how heterointerface engineering governs photocatalytic activity, ROS selectivity, mineralization efficiency, and operational stability. Unlike previous reviews focused primarily on antibacterial activity or generalized photocatalytic systems, the present work specifically examines antibiotic degradation as a sustainable strategy for mitigating AMR-related environmental risks. Current limitations, including insufficient mechanistic understanding, lack of standardized benchmarking protocols, limited real-water validation, and incomplete long-term environmental safety assessment, are critically discussed together with future research directions toward scalable and environmentally responsible photocatalytic technologies.

## 2 Fundamentals of TiO_2_ and carbon dots

A fundamental understanding of the structural, electronic, and surface properties of TiO_2_ and CDs is essential for interpreting the photocatalytic behavior of CDs/TiO_2_ nanocomposites. The performance of these hybrid systems is governed not only by heterojunction formation, but also by the intrinsic band structure, defect states, surface chemistry, and charge-transfer characteristics of each component. Therefore, this section summarizes the key physicochemical properties of TiO_2_ and CDs that govern interfacial coupling and photocatalytic antibiotic degradation.

### 2.1 TiO_2_: crystal structure and photocatalytic principles

TiO_2_ exists primarily in three crystalline polymorphs: anatase, rutile, and brookite, as illustrated in Fig. 3.^43^ These polymorphs differ in lattice symmetry, crystal structure, density, and electronic properties, which collectively influence photocatalytic performance. Anatase is generally considered the most photocatalytically active phase because of its relatively slow electron–hole recombination kinetics, higher surface area, and favorable conduction-band position for oxygen reduction reactions.^44–46^ Rutile, although thermodynamically more stable, often exhibits lower photocatalytic activity because of faster charge recombination.^47,48^ Brookite has received comparatively less attention because of difficulties associated with phase-pure synthesis and its lower thermodynamic stability relative to rutile.^48,49^

**Fig. 3:**
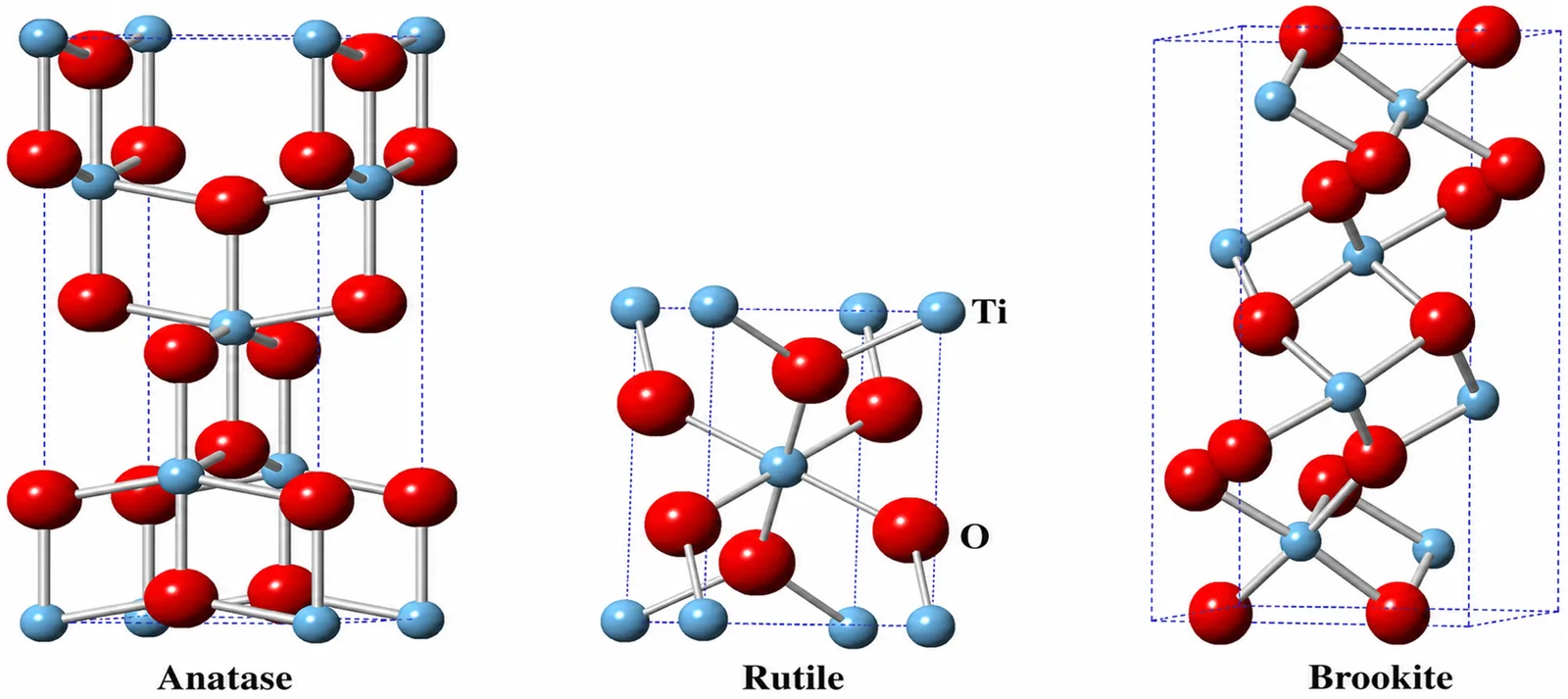
Schematic illustration of the crystal structures of the three major TiO_2_ polymorphs: anatase, rutile, and brookite. Reproduced with permission from ref. 43 copyright 2015, Elsevier.

Upon irradiation with photons possessing energy equal to or greater than the TiO_2_ bandgap, photogenerated electrons are excited from the valence band to the conduction band, leaving positively charged holes in the valence band.^50,51^ For anatase and rutile TiO_2_, the bandgap energies are approximately 3.2 and 3.0 eV, respectively. The resulting charge carriers subsequently migrate toward the catalyst surface and participate in interfacial redox reactions.^52^

Conduction-band electrons reduce dissolved oxygen to generate superoxide radicals (O_2_˙^−^), whereas valence-band holes oxidize water molecules or hydroxide ions to form hydroxyl radicals (˙OH).^53,54^ These reactive oxygen species (ROS) possess sufficiently strong oxidation potentials to degrade persistent organic contaminants, including antibiotic molecules containing aromatic and heterocyclic structures.^55,56^

Despite these advantages, pristine TiO_2_ suffers from two major intrinsic limitations. First, rapid bulk and surface recombination of photogenerated charge carriers substantially reduces quantum efficiency and limits ROS generation.^57,58^ Second, the wide bandgap of TiO_2_ restricts photoexcitation primarily to the ultraviolet region, which represents only a small fraction of the solar spectrum.^23,50^ Consequently, extensive efforts have focused on improving visible-light responsiveness and charge separation through doping, heterojunction engineering, and incorporation of conductive carbonaceous materials.^59,60^

### 2.2 Carbon dots: structure, classification, and properties

CDs represent a family of quasi-spherical, zero-dimensional carbon nanomaterials typically smaller than 10 nm in diameter.^61,62^ Since their discovery, CDs have evolved into a versatile class of carbon-based nanostructures that includes carbon quantum dots (CQDs), graphene quantum dots (GQDs), and polymer-derived CDs, which are generally classified according to their structural characteristics and synthetic origin.^63,64^

CDs can be synthesized through either top-down or bottom-up approaches. Top-down methods involve fragmentation of bulk carbon materials using techniques such as laser ablation, arc discharge, electrochemical exfoliation, or chemical oxidation. In contrast, bottom-up approaches rely on carbonization of molecular precursors or biomass-derived feedstocks through hydrothermal, solvothermal, microwave-assisted, or pyrolytic synthesis routes.^65–67^ The selected synthetic strategy strongly influences particle size, crystallinity, heteroatom incorporation, surface-state distribution, and functional-group density.^68,69^

Structurally, CDs consist of nanoscale sp^2^-conjugated carbon domains embedded within an amorphous sp^3^-hybridized carbon matrix and surrounded by oxygen- or nitrogen-containing surface functional groups, including hydroxyl, carboxyl, carbonyl, and amine moieties.^70–72^ As illustrated in Fig. 4, this core–shell-like architecture plays a critical role in determining their optical and electronic properties. These surface functionalities improve aqueous dispersibility, regulate surface charge, and provide active anchoring sites for coupling with semiconductor surfaces.^73,74^

**Fig. 4:**
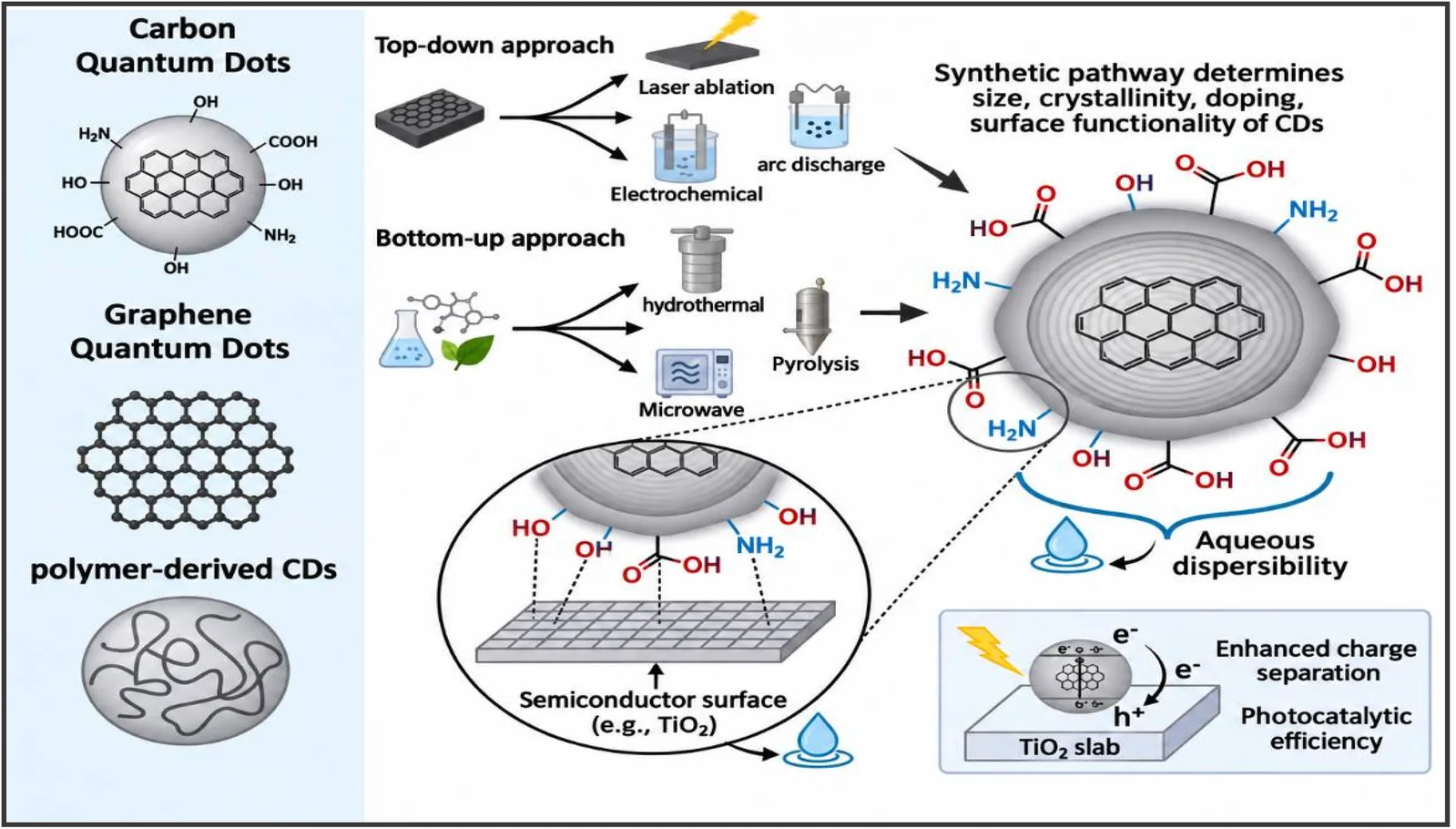
Schematic illustration of structural and optical characteristics of CDs, including core–shell architecture, representative top-down and bottom-up synthesis approaches, and surface functional groups. The tunable electronic structure of CDs enables visible-light absorption and interfacial charge-transfer functionality.

The electronic and optical properties of CDs strongly depend on particle size, graphitization degree, heteroatom doping, and surface-state distribution.^75,76^ Their tunable band structure enables efficient visible-light absorption, adjustable work-function alignment, and effective interfacial electron transfer.^36,39^ Consequently, CDs may function as electron donors, electron acceptors, photosensitizers, or conductive mediators depending on the energetic configuration of the composite system.^77,78^

Heteroatom doping, especially with nitrogen and sulfur, effectively modulates the electronic structure of CDs and reduces their work function, thereby promoting visible-light absorption and facilitating interfacial charge transfer.^79,80^ In addition, CDs exhibit characteristic photoluminescence behavior originating from quantum confinement effects and surface-state emissions.^81^ Although some studies have proposed that CDs exhibit up-conversion photoluminescence (UCPL), enabling excitation by lower-energy light followed by the emission of higher-energy photons causing an anti-Stokes shift, the practical contribution of this mechanism remains under debate. The most commonly suggested mechanism involves the excitation of electrons from the π orbital to higher-energy states through the absorption of multiple low-energy photons, followed by their return to the σ orbital *via* radiative relaxation.^32^

Importantly, the ability of CDs to transiently store and shuttle electrons makes them highly effective for suppressing charge recombination in semiconductor photocatalysts.^82,83^ Through rational control of surface chemistry and electronic structure, CDs can significantly influence charge separation efficiency, ROS generation, and adsorption behavior in photocatalytic systems.^84^

### 2.3 Complementary properties enabling synergistic photocatalysis

The integration of CDs with TiO_2_ is fundamentally driven by complementary electronic structures and surface functionalities that collectively improve light harvesting, interfacial charge transfer, and ROS-mediated oxidation efficiency.^85,86^ TiO_2_ provides excellent physicochemical stability and efficient ROS-generation capability, whereas CDs contribute tunable electronic states, visible-light responsiveness, electron transfer, and storage functionality.^87,88^

Strong interfacial coupling between CDs and TiO_2_, frequently involving Ti–O–C linkages, facilitates efficient electronic communication across the heterointerface.^89,90^ This interfacial interaction promotes directional charge migration, suppresses electron–hole recombination, and improves the separation efficiency of photogenerated carriers.^91^ Simultaneously, the π-conjugated domains of CDs can enhance adsorption of aromatic antibiotic molecules through π–π interactions, thereby increasing local reactant concentration at photocatalytic sites.^92,93^

In addition to electronic modulation, the surface functional groups and π-conjugated structure of CDs provide additional active sites for adsorption and increase the surface area when incorporated with TiO_2_, as illustrated in Fig. 5.^94^ Oxygen vacancies and defect states, together with CDs/TiO_2_, further improve photocatalytic activity and ROS formation.^95,96^ Consequently, the synergistic integration of CDs and TiO_2_ establishes the foundation for efficient visible-light-responsive photocatalytic systems.^97^

**Fig. 5:**
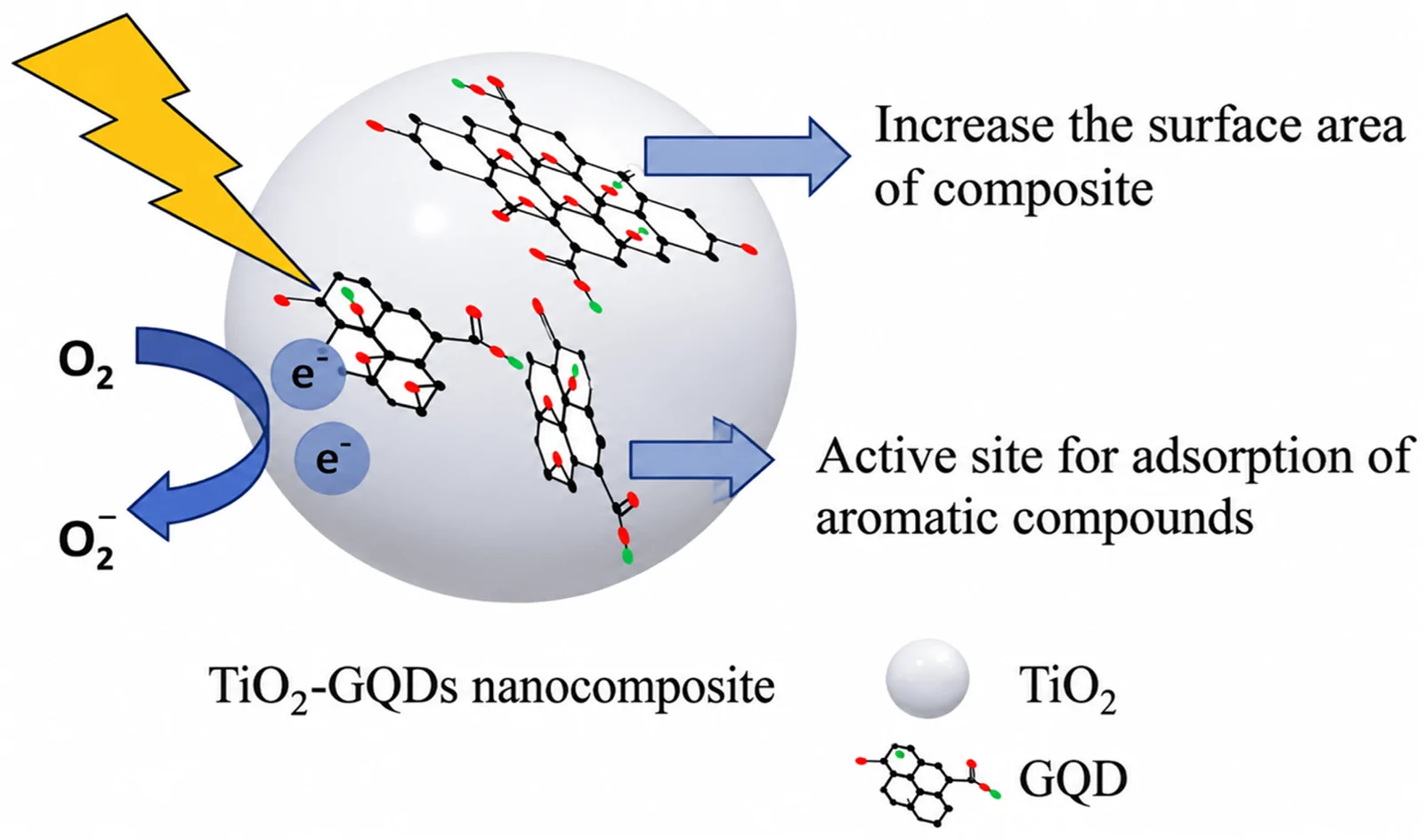
Schematic representation of the enhancement of surface area of TiO_2_ due to GQD nanoparticles. Adapted with permission from ref. 94 copyright 2021, MDPI.

Collectively, the complementary optical, electronic, and surface properties of TiO_2_ and CDs provide the basis for heterointerface engineering in hybrid photocatalysts. These interfacial interactions govern charge-transfer pathways, ROS generation, and ultimately the photocatalytic degradation efficiency of antibiotic pollutants.^98,99^

## 3 Interfacial engineering in CDs/TiO_2_ nanocomposites

The enhanced photocatalytic performance of CDs/TiO_2_ nanocomposites originates primarily from the formation of electronically coupled heterointerfaces rather than simple compositional hybridization. These interfacial regions regulate charge-carrier migration, band alignment, adsorption behavior, defect-state distribution, and ROS generation, thereby governing the overall photocatalytic efficiency toward antibiotic degradation. Consequently, rational interfacial engineering has become a central strategy for improving visible-light responsiveness, suppressing electron–hole recombination, and enhancing photocatalytic selectivity in CDs/TiO_2_ systems.

### 3.1 Interfacial chemical bonding and structural coupling

Efficient electronic communication between CDs and TiO_2_ requires intimate interfacial contact. In hydrothermal and solvothermal synthesis systems, hydroxyl groups on TiO_2_ surfaces can react with oxygen-containing functional groups on CDs, particularly carboxyl and hydroxyl moieties, leading to the formation of Ti–O–C interfacial linkages.^100,101^ As illustrated in Fig. 6a, condensation reactions between TiO_2_ surface hydroxyl groups and functionalized CDs generate chemically bonded heterointerfaces accompanied by water elimination.^100^ CDs can also interact with TiO_2_ through physical mixing, which involves blending the two components by stirring or ultrasonication, followed by their assembly through noncovalent interactions. Although this approach is facile and rapid, the interaction between CDs and TiO_2_ achieved by physical mixing is generally weak and unstable, which may limit efficient interfacial charge separation and transfer.^102^

**Fig. 6:**
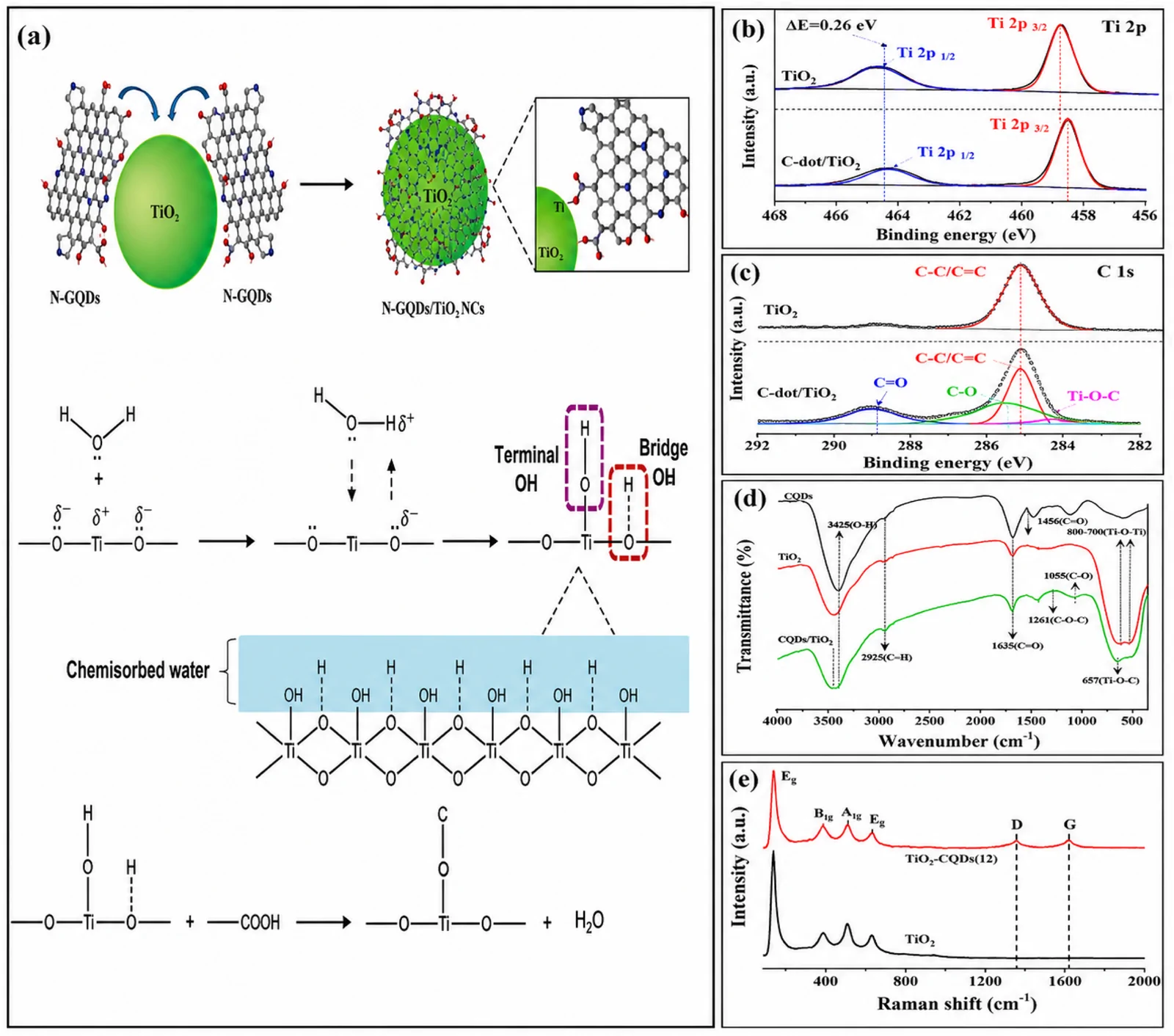
Spectroscopic and structural evidence of interfacial chemical coupling in CDs/TiO_2_ nanocomposites. (a) Schematic of Ti–O–C bond formation *via* condensation between TiO_2_ surface hydroxyl groups and oxygen-containing functionalities on CDs. Adapted with permission from ref. 100 copyright 2020, Royal Society of Chemistry. (b and c) XPS core-level spectra showing binding-energy shifts for (b) Ti 2p and (c) C 1s. The C 1s peaks at 288.8, 285.2, and 284.1 eV correspond to C=O, C–O, and Ti–O–C, respectively, indicating chemical interaction between TiO_2_ and carbon domains. Adapted with permission from ref. 103 copyright 2023, American Chemical Society. (d) FTIR spectrum revealing vibrational signatures characteristic of Ti–O–C interfacial linkages. Adapted with permission from ref. 104 copyright 2025, Elsevier. (e) Raman spectra displaying the anatase TiO_2_ lattice modes alongside the D and G bands of graphitic carbon, confirming the coexistence and coupling of both components. Adapted with permission from ref. 105 copyright 2014, Elsevier.

Multiple spectroscopic techniques provide evidence for strong interfacial coupling. X-ray photoelectron spectroscopy (XPS) commonly reveals shifts in Ti 2p binding energies, and C 1s peaks were located at 288.8, 285.2, and 284.1 eV, which correspond to C=O, C–O, and Ti–O–C (Fig. 6b and c).^103^ Fourier-transform infrared (FTIR) spectroscopy identifies vibrational features associated with Ti–O–C bonding (Fig. 6d).^104^ Whereas Raman spectroscopy confirms preservation of TiO_2_ lattice modes together with the appearance of D and G bands characteristic of graphitic carbon domains (Fig. 6e).^105^ Collectively, these observations demonstrate that strong interfacial coupling facilitates directional charge transfer and efficient electronic communication across the heterointerface.^106,107^

In addition to improving charge-transfer efficiency, strong chemical anchoring contributes to structural stability during photocatalytic cycling by minimizing catalytic leaching and preserving interfacial conductivity under irradiation conditions.^108,109^ However, excessive carbon loading may shield TiO_2_ active sites, reduce photon penetration, or introduce additional recombination centers, ultimately decreasing photocatalytic activity.^110,111^ Therefore, optimization of interfacial coverage is essential to balance electronic conductivity with surface accessibility.

### 3.2 Band alignment and heterojunction architectures

The photocatalytic behavior of CDs/TiO_2_ nanocomposites is fundamentally governed by interfacial band alignment and charge-transfer pathways. Semiconductor heterojunctions are formed by integrating two semiconductors with different band gap energies, while the combination of multiple heterojunctions leads to the formation of heterostructures. In this context, the integration of carbon dots (CDs) with TiO_2_ has emerged as an effective strategy for photocatalytic degradation by overcoming the intrinsic limitations of pristine TiO_2_. The specific heterojunction architecture formed between CDs and TiO_2_ plays a decisive role in regulating charge migration, separation efficiency, and overall photocatalytic performance.^112^ Depending on the interfacial structure and electronic interactions between CDs and TiO_2_, various charge-transfer configurations, including Schottky junctions, type II heterojunctions, direct Z-scheme, as shown in Fig. 7a,^112^ and S-scheme heterojunctions, can be formed.

**Fig. 7:**
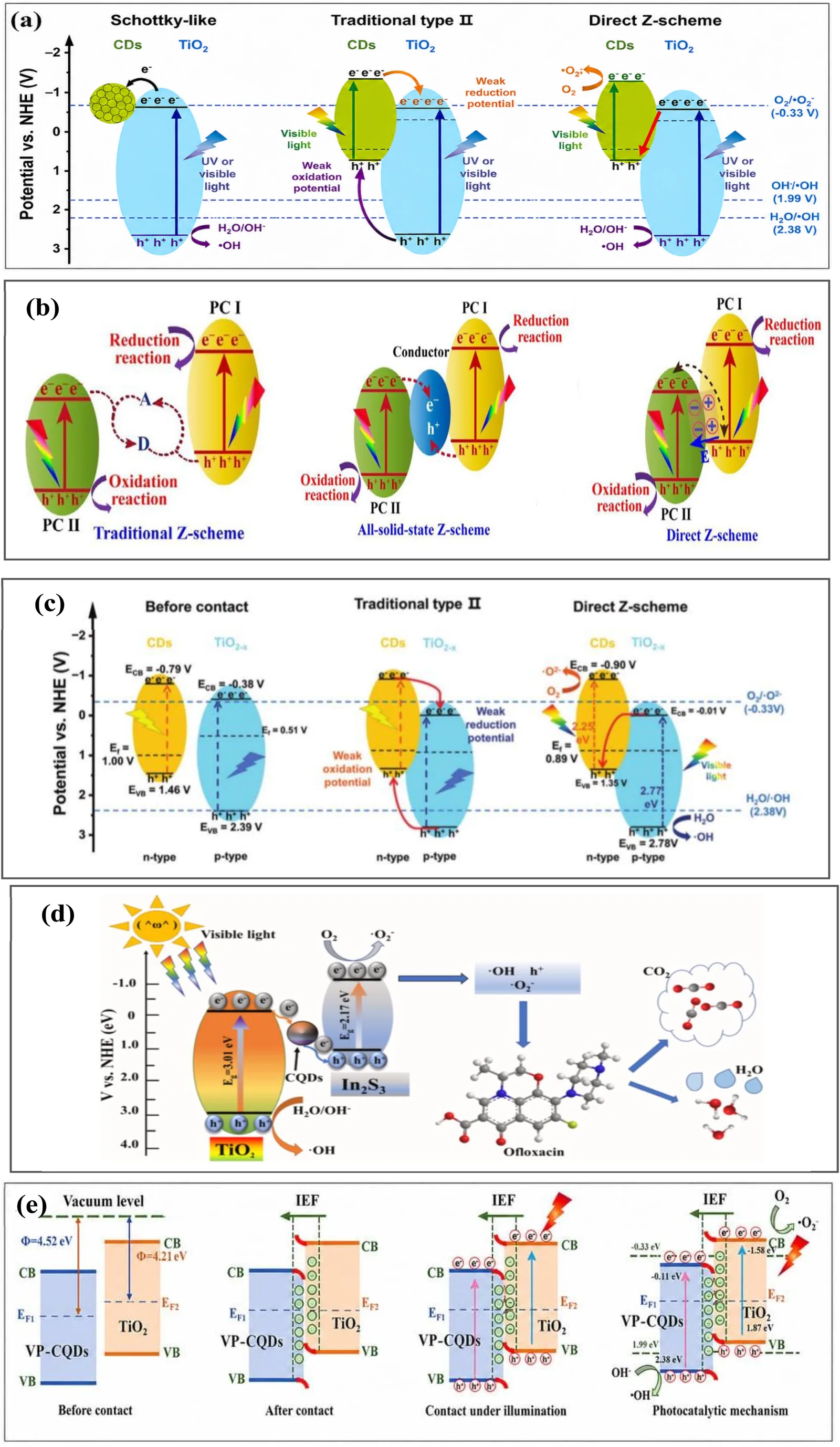
(a) Schematic representations of heterojunction architectures in CDs/TiO_2_ photocatalytic systems, band alignment, and charge-transfer pathways for Schottky-like, type II, and direct Z-scheme configurations. Adapted with permission from ref. 112 copyright 2025, Elsevier. (b) Schematic illustration of charge carrier transfer in different types of Z-scheme photocatalysts. Adapted with permission from ref. 114 copyright 2017, Elsevier. (c) Energy-band structures and plausible mechanisms of electron transfer for the CDs@TiO_2−*x*_ heterojunctions. Adapted with permission from ref. 115 copyright 2024, Wiley. (d) The photocatalytic mechanism of degradation of OFL by the all-solid-state Z-scheme In_2_S_3_/CQDs/TiO_2_ heterojunction. Adapted with permission from ref. 117 copyright 2022, Elsevier. (e) The energy band arrangements of VP-CQDs and TiO_2_ before contact, after contact in darkness, contact under illumination, and photocatalytic mechanism. Adapted with permission from ref. 109 copyright 2026, Elsevier.

In a traditional type II heterojunction, the two semiconductor components possess a staggered band alignment. Upon photoexcitation, photogenerated electrons migrate from the conduction band (CB) of semiconductor I to the CB of semiconductor II, while photogenerated holes migrate from the valence band (VB) of semiconductor II to the VB of semiconductor I. This directional charge transfer spatially separates the photogenerated carriers and effectively suppresses electron–hole recombination. However, this configuration remains fundamentally limited in redox ability. Because the transferred electrons accumulate in the CB of semiconductor II with a weaker reduction potential, while the transferred holes accumulate in the VB of semiconductor I with a weaker oxidation potential.^113^

In a direct Z-scheme heterojunction, the charge-transfer pathway is determined by the band alignment and interfacial electronic interactions between the two semiconductors. Upon light irradiation, photoexcited electrons in the conduction band (CB) of the oxidation photocatalyst (PC II) recombine directly with holes in the valence band (VB) of the reduction photocatalyst (PC I) through the interface. This selective recombination eliminates charge carriers with weaker redox abilities, while preserving electrons with strong reduction ability in the CB of PC I and holes with strong oxidation ability in the VB of PC II. As a result, the direct Z-scheme system achieves efficient charge separation while maintaining strong redox potentials for photocatalytic reactions (Fig. 7b). Schematic illustration of charge carrier transfer in different types of Z-scheme photocatalysts.^114^ Cong *et al.*^115^ fabricated a xylan-based carbon dots@hydrogenated TiO_2_ (CDs@TiO_2−*x*_) nanocomposite through the construction of a direct Z-scheme heterojunction. Under light irradiation, the direct Z-scheme pathway allowed electrons in the conduction band of TiO_2−*x*_ to recombine with holes in the valence band of CDs, preserving highly reductive electrons on the CDs and strongly oxidative holes on TiO_2−*x*_ for photocatalytic reactions (Fig. 7c). As a result, the CDs@TiO_2−*x*_ composite exhibited enhanced visible-light photocatalytic performance, achieving 89%, 87% degradation of methylene blue, methyl orange respectively within 90 min, as well as 99% rhodamine B degradation within 60 min. Xu *et al.*^116^ constructed a TiO_2_/CQD Z-scheme heterojunction through simple mechanical mixing driven by electrostatic attraction between positively charged TiO_2_ and negatively charged CQDs. This interfacial interaction promoted electron transfer and generated an internal electric field, facilitating the separation and migration of photogenerated charge carriers. In the proposed Z-scheme pathway, electrons in the TiO_2_ conduction band recombined with holes in the CQD valence band, preserving highly reactive electrons on CQDs and holes on TiO_2_ for photocatalytic reactions. The resulting composite achieved about 98% tetracycline degradation within 30 min and maintained good catalytic activity after four cycles, indicating favorable stability and reusability. Most studies on CDs/TiO_2_ heterojunctions use CDs as electron mediators in all-solid-state Z-scheme systems, whereas direct Z-scheme CDs/TiO_2_ heterojunctions remain less explored. Ding *et al.*^117^ reported that CQDs served as an electron mediator in the In_2_S_3_/CQDs/TiO_2_ all-solid-state Z-scheme heterojunction, accelerating interfacial charge transfer and promoting efficient charge separation. By regulating the electron migration pathway, CQDs preserved the strong reduction ability of electrons in the In_2_S_3_ conduction band and the strong oxidation ability of holes in the TiO_2_ valence band, resulting in enhanced photocatalytic performance (Fig. 7d). The composite achieved 92.7% degradation of ofloxacin within 120 min under simulated sunlight irradiation. Qu *et al.*^118^ constructed an N, S-CQDs/Bi_2_MoO_6_@TiO_2_ Z-scheme heterojunction, where N, S-CQDs acted as an electron-transfer mediator to facilitate interfacial charge migration. The introduction of N, S-CQDs improved visible-light absorption and promoted the separation and transfer efficiency of photogenerated carriers, leading to enhanced photocatalytic performance. The authors attributed the improved activity to the synergistic effect of surface-functionalized N, S-CQDs and the Z-scheme charge-transfer system, which achieved 85.8% degradation of ciprofloxacin within 240 min under visible-light irradiation.

More recently, S-scheme heterojunctions have attracted increasing attention because they combine efficient charge separation with strong oxidation and reduction capabilities through the formation of an internal electric field and interfacial band bending.^119^ The S-scheme can be considered a refinement of the direct Z-scheme concept, in which the internal electric field and band bending are explicitly considered as driving forces for the selective recombination of low-energy carriers. Meng *et al.*^109^ constructed a VP-CQDs/TiO_2_ S-scheme heterojunction by coupling *Viola philippica*-derived carbon quantum dots with TiO_2_ through an impregnation–calcination method. The difference in work functions between TiO_2_ and VP-CQDs induced spontaneous interfacial electron transfer, leading to Fermi-level equilibration, band bending, and the formation of an internal electric field from TiO_2_ toward VP-CQDs. Under light irradiation, electrons in the conduction band of VP-CQDs recombined with holes in the valence band of TiO_2_, while highly reducing electrons remained in the TiO_2_ conduction band and strongly oxidizing holes were retained in the VP-CQD valence band (Fig. 7e). This S-scheme charge-transfer pathway promoted efficient separation and migration of photogenerated carriers while preserving strong redox capability, thereby facilitating the generation of ˙O_2_^−^ and ˙OH radicals for pollutant degradation. The optimized 3-VP-CQDs/TiO_2_ composite achieved 97.34% tetracycline and 96.80% malachite green degradation within 120 min.

In some composite systems, CDs may exhibit Schottky-like behavior, serving as electron reservoirs that capture and shuttle photogenerated electrons away from TiO_2_, thereby suppressing interfacial recombination. However, explanations using Schottky-like junctions lack sufficient scientific rigor, as it primarily emphasizes the electron-transfer ability of CDs while ignoring their complex band structures and surface functional groups.^112^ And in type II heterojunctions, photogenerated electrons and holes migrate toward different semiconducting regions, improving spatial charge separation but often weakening overall redox capability. Direct Z-scheme and S-scheme can solve the problem of carrier recombination while maintaining strong redox ability.^120^

Despite extensive mechanistic discussion in the literature, direct experimental determination of interfacial band alignment remains relatively limited.^121,122^ Heng *et al.*^123^ proposed that the NCQDs/TiO_2_ system for methylene blue degradation followed a direct Z-scheme mechanism rather than a type-II heterojunction, based on electron scavenger experiments that demonstrated the formation of both ˙O_2_^−^ and ˙OH radicals. In a conventional type-II mechanism, the remaining electrons on the TiO_2_ conduction band and holes on the NCQDs valence band would not possess sufficient redox potentials to produce these radicals. However, in the direct Z-scheme pathway, electrons remain on the conduction band of NCQDs and holes remain on the valence band of TiO_2_, enabling the formation of ˙O_2_^−^ and ˙OH radicals. Therefore, the scavenging results together with the band-edge potential analysis supported the direct Z-scheme charge-transfer mechanism as shown in Fig. 8a and b. Although radical trapping experiments, electron spin resonance (ESR), and photoluminescence are widely used to investigate charge-transfer mechanisms, these methods rely on the detection of reactive oxygen species (ROS) and require accurate determination of band positions and redox potentials to interpret possible reaction pathways. However, literature reviews emphasize that complementary techniques, particularly *in situ* XPS, represent the most direct approaches for providing evidence of interfacial charge-transfer processes and distinguishing different heterojunction mechanisms.^124^ Cong *et al.*^115^ confirmed the direct Z-scheme mechanism of CDs@TiO_2−*x*_ through complementary band-structure analysis, EPR, and *in situ* XPS. The band positions indicated that a conventional type-II pathway could not provide sufficient redox potentials for the observed formation of ˙O_2_^−^ and ˙OH, while EPR directly verified these reactive species. To provide more conclusive evidence, *in situ* XPS was performed under illumination, revealing a shift of C 1s toward lower binding energy and Ti 2p and O 1s toward higher binding energy. These shifts indicated photoelectron transfer from TiO_2−*x*_ to CDs, consistent with the proposed direct Z-scheme charge-transfer pathway as shown in Fig. 9.

**Fig. 8:**
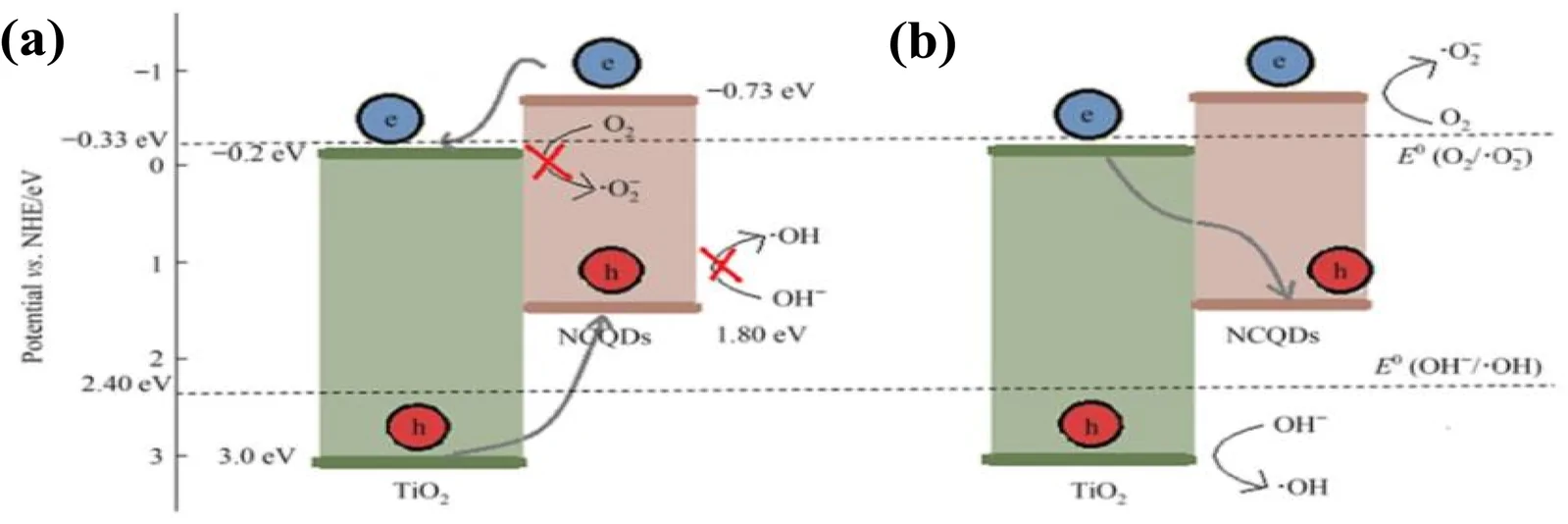
The proposed charge-transfer mechanisms of (a) type-II and (b) direct Z-scheme heterojunctions for NCQDs/TiO_2_ photocatalysts. Adapted with permission from ref. 123 copyright 2022, Elsevier.

**Fig. 9:**
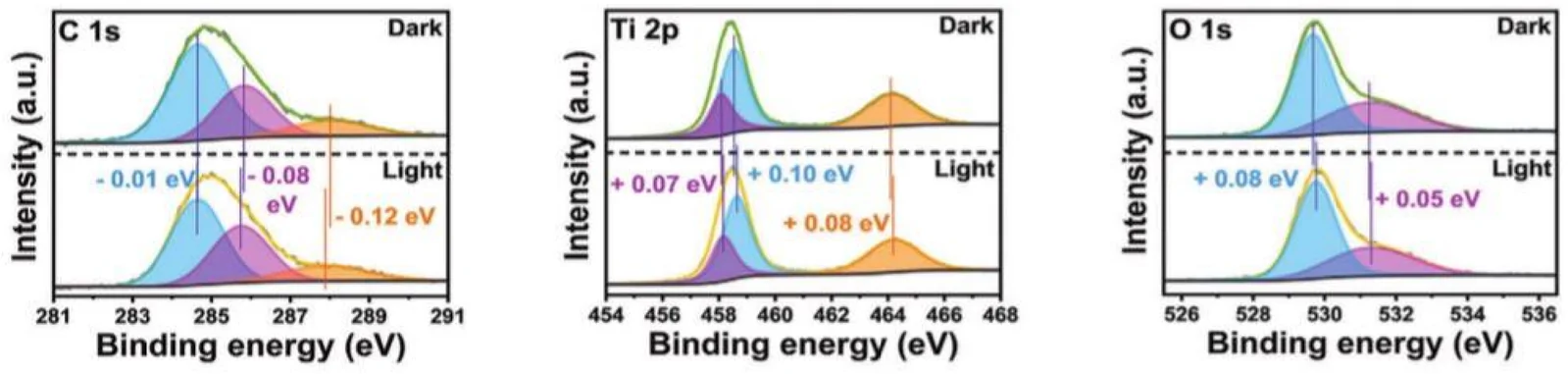
High-resolution C 1s, Ti 2p, and O 1s spectra without and with irradiation of CDs@TiO_2−*x*_. Adapted with permission from ref. 115 copyright 2024, Wiley.

### 3.3 Charge carrier dynamics at the heterointerface

Charge separation and recombination dynamics strongly influence photocatalytic efficiency in CDs/TiO_2_ systems. Incorporation of CDs frequently results in substantial photoluminescence quenching, indicating suppression of radiative electron–hole recombination (Fig. 10a).^125^ Time-resolved photoluminescence studies further demonstrate prolonged carrier lifetimes in CD-modified TiO_2_ compared with pristine TiO_2_. The longer average PL decay lifetime indicates that the photogenerated charge carriers persist for a longer period before recombination, providing kinetic evidence for delayed carrier recombination in the CD-modified system (Fig. 10b).^126^

**Fig. 10:**
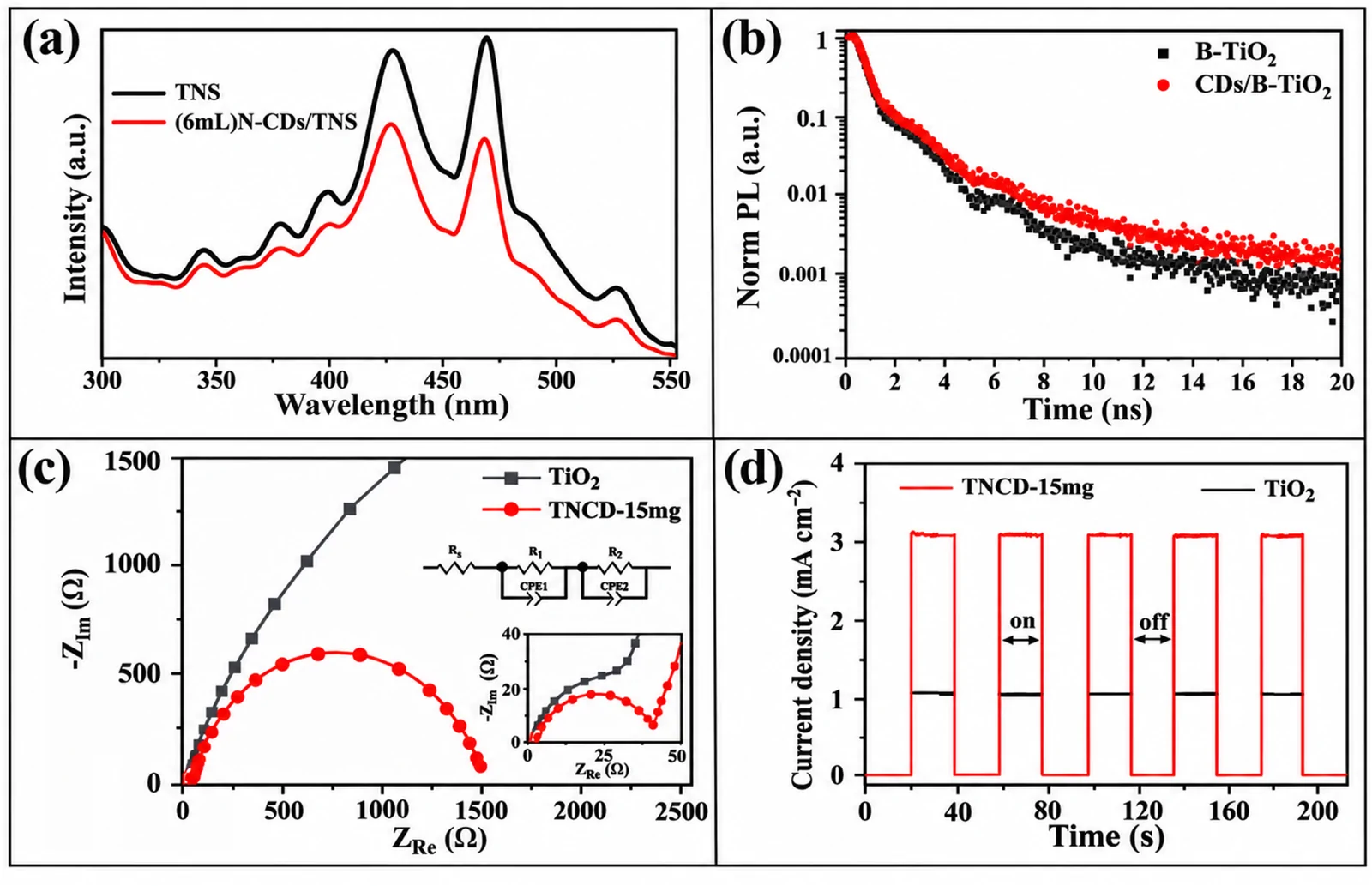
Spectroscopic and electrochemical characterization of charge-carrier dynamics in CDs/TiO_2_ nanocomposites. (a) Photoluminescence (PL) spectra showing significant emission quenching upon CD incorporation. Adapted with permission from ref. 125 copyright 2019, Royal Society of Chemistry. (b) Time-resolved photoluminescence (TRPL) decay profiles revealing prolonged carrier lifetimes in CDs/B-TiO_2_ compared to pristine B-TiO_2_, reflecting enhanced charge separation. Adapted with permission from ref. 126 copyright 2023, American Chemical Society. (c) Nyquist plots from electrochemical impedance spectroscopy (EIS) demonstrating reduced charge-transfer resistance at the electrode–electrolyte interface after CD functionalization. (d) Transient photocurrent response curves showing improved photogenerated carrier mobility and interfacial charge-transfer efficiency under intermittent illumination. Panels (c and d) adapted with permission from ref. 127 copyright 2022, Elsevier.

Electrochemical impedance spectroscopy (EIS) commonly reveals reduced charge-transfer resistance after CD incorporation, indicating effective charge-separation behavior (Fig. 10c). Meanwhile, transient photocurrent measurements show that pristine TiO_2_ exhibits a lower photocurrent density under light irradiation, consistent with faster electron–hole recombination. In contrast, incorporation of CDs increases the photocurrent density due to suppressed charge recombination (Fig. 10d).^127^ These spectroscopic and electrochemical observations collectively demonstrate that rational interfacial engineering suppresses recombination, prolongs carrier lifetime, and increases the probability of ROS-generating surface reactions.^128^

Extended carrier lifetime is particularly important because it increases the likelihood of photogenerated electrons and holes reaching surface-active sites before recombination occurs. Consequently, efficient interfacial charge separation directly contributes to improved photocatalytic degradation performance.^129^

### 3.4 Light-harvesting enhancement mechanisms

Integration of CDs significantly broadens the optical response of TiO_2_ through sensitization and interfacial electron-transfer mechanisms.^130^ Under visible-light irradiation, photoexcited electrons in the CDs can be injected into the conduction band of TiO_2_, where they facilitate the reduction of dissolved O_2_ and subsequently promote the generation of ROS.^99,131^

As illustrated in Fig. 11a and b, interfacial Ti–O–C bonding facilitates directional electron transfer from GQDs toward TiO_2_, improving charge separation efficiency and enhancing photocatalytic oxidation activity.^132^ In this sensitization process, GQDs act as visible-light absorbers and electron donors that extend the effective photoresponse of TiO_2_ into the visible region.

**Fig. 11:**
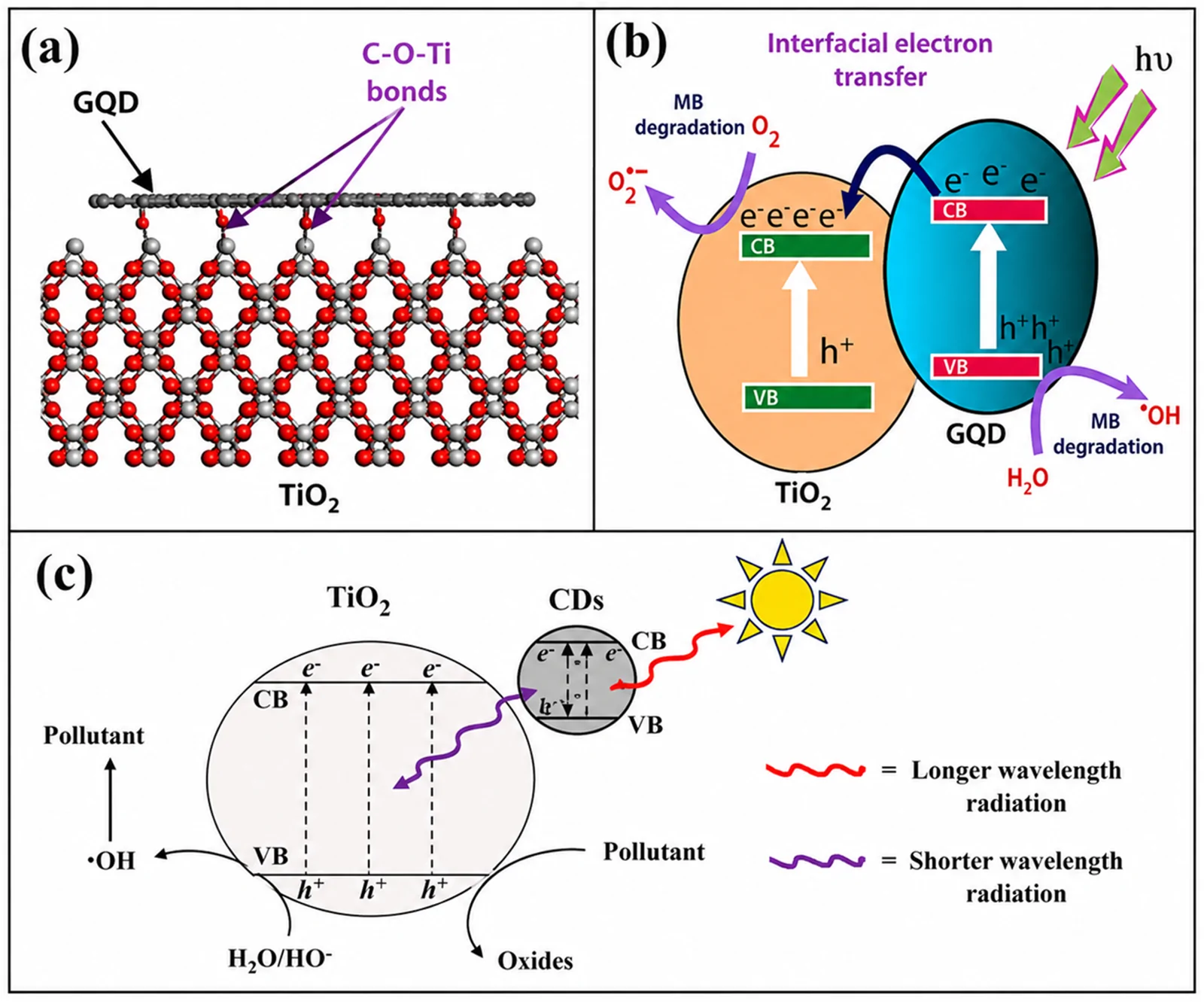
Light-harvesting enhancement mechanisms in CDs/TiO_2_ nanocomposites. (a) Schematic of Ti–O–C interfacial bonding formed between GQDs and the TiO_2_ surface, establishing an electronic bridge for charge transfer; (b) visible-light-driven sensitization process wherein photoexcited electrons from GQDs are injected into the TiO_2_ conduction band, enabling subsequent oxygen reduction and reactive oxygen species (ROS) generation. Adapted with permission from ref. 132 copyright 2017, Elsevier. (c) Proposed up-conversion photoluminescence (UCPL) mechanism, illustrating the absorption of lower-energy photons by CDs and subsequent emission of higher-energy photons that potentially excite TiO_2_. Adapted with permission from ref. 32 copyright 2022, Elsevier.

Some studies have additionally proposed that CDs exhibit up-conversion photoluminescence (UCPL), in which lower-energy photons generate higher-energy emissions capable of indirectly exciting TiO_2_. Fig. 11c schematically illustrates this proposed mechanism.^32^ However, the practical contribution of UCPL to photocatalytic enhancement remains controversial.^133,134^ Therefore, visible-light sensitization and interfacial electron transfer are generally considered the dominant mechanisms responsible for photocatalytic enhancement in CDs/TiO_2_ systems, while up-conversion photoluminescence (UCPL) has also been proposed as a light-harvesting pathway in some studies.

### 3.5 Interface-driven surface reactivity

Interfacial engineering not only modifies electronic structure but also strongly influences adsorption and photocatalytic activities.^135^ Incorporation of CDs can modulate the concentration of oxygen vacancies in TiO_2_ through interfacial electronic interactions.^96^ These defect sites facilitate oxygen adsorption and promote superoxide radical formation by enhancing electron capture by oxygen molecules.^136,137^

Surface functional groups on CDs, including hydroxyl, carboxyl, carbonyl, and amine moieties, additionally regulate adsorption affinity toward antibiotic molecules through electrostatic attraction, hydrogen bonding, and π–π interactions.^79,138^ Enhanced adsorption increases the availability of pollutant molecules at photocatalytically active surface sites, thereby improving accessibility of ROS to target molecules and accelerating oxidative degradation reactions.^139,140^

Collectively, the photocatalytic behavior of CDs/TiO_2_ nanocomposites emerges from the synergistic interaction between interfacial charge transfer, enhanced adsorption, defect-state engineering, and ROS generation.^135,141^ Therefore, rational tuning of heterointerface properties is essential for maximizing photocatalytic antibiotic degradation efficiency under environmentally relevant conditions.

## 4 Mechanistic pathways of antibiotic degradation

The photocatalytic degradation of antibiotics over CDs/TiO_2_ nanocomposites proceeds through a coupled sequence of photophysical excitation, interfacial charge transfer, ROS generation, molecular oxidation, and progressive mineralization. Unlike pristine TiO_2_, incorporation of carbon dots introduces electronically integrated heterointerfaces that regulate carrier lifetime, visible-light responsiveness, and oxygen activation, thereby strongly influencing degradation selectivity and efficiency. Consequently, photocatalytic degradation performance is governed by the interplay between interfacial electronic structure, ROS distribution, adsorption equilibrium, and antibiotic molecular properties. Representative CDs/TiO_2_ photocatalytic systems, interfacial configurations, and degradation outcomes reported in the literature are summarized in Table 1.

**Table 1:** Representative CDs/TiO_2_ heterojunctions for photocatalytic antibiotic degradation: synthesis strategy, interfacial characteristics, dominant reactive species, and photocatalytic performance

CDs precursor (synthesis route)	TiO_2_ phase (composed synthesis)	Interface nature (heterojunction)	Role of CDs	Dominant reactive species (optimum condition)	Key photocatalytic outcome	Ref.
*Rumex crispus* L. plant + urea (hydrothermal)	Anatase (hydrothermal)	Ti–O–C chemical bonding (S scheme heterojunction)	Electron acceptor and carrier	˙O_2_^−^ ≈ h^+^ > ˙OH (10 mg per L TC, pH 5.15, and 0.2 g per L catalyst)	97.73% TC degradation in 120 min; under UVA-light	79
l-Ascorbic acid (hydrothermal)	Anatase (hydrothermal + stirring)	TiO_2_/C-dots nanoparticles assembled on Bi_2_WO_6_ nanoflakes (Z-scheme heterojunction)	Electron reservoir, upconversion PL	e^−^ and h^+^ (10 mg per L levofloxacin, pH 7, and 0.075 g per L catalyst)	99% levofloxacin degradation in 90 min under solar light	121
Chitosan + urea (one-step hydrothermal carbonization)	Anatase (hydrothermal)	Strong chemical coupling *via* Ti–O–C (S-scheme heterojunction)	Electron acceptors and donors	h^+^ > ˙OH_surface_ > ˙O_2_^−^ (10 mg per L CIP, pH 5, and 0.4 g per L catalyst)	83.91% CIP degradation in 120 min under UVA light	168
Citric acid + urea (microwave)	Anatase (hydrothermal, ultrasonication, and stirring)	Strong interfacial interaction between TiO_2_ and NCDs/TCN (direct Z-type heterojunction)	Interfacial electron transport bridge, photosensitizers, up conversion	˙O_2_^−^ > h^+^ > ˙OH (20 mg per L LOFL, pH 7, 0.8 g per L catalyst)	89.6% LOFL degradation in 80 min under visible light	169
Citric acid + urea (vacuum heating)	Anatase (electrostatic mixing)	Electrostatic attraction (Z-scheme heterojunction)	Accelerates e^−^/h^+^ separation and transfer, up conversion	˙O_2_^−^ ≈ h^+^ > ˙OH (20 mg per L TC, 0.5 g per L catalyst, 9 wt% CQDs/TiO_2_)	98% TC degradation in 30 min under visible light	116
Viola philippica leaves (one-step hydrothermal)	Anatase (impregnation–calcination)	Strong chemical interface with Ti–O–C bonding (S-scheme heterojunction)	Electron acceptor, facilitates electron transfer	˙O_2_^−^ > ˙OH > h^+^ > ^1^O_2_ (3-VP-CQDs/TiO_2_, 10 mg per L TC, 1.5 g per L catalyst, pH 8)	97.34% TC degradation in 120 min under simulated light	109
Citric acid + urea (hydrothermal)	P25 anatase/rutile (mixing and stirring)	CDs embedded in TiO_2_ and deposited on g-C_3_N_4_ surface (Z-scheme heterojunction)	Electron mediators, upconversion PL	˙O_2_^−^ > ˙OH > h^+^, ^1^O_2_ (4 mg per L ENX, 1.0 g per L catalyst, 1.0 wt% CDs, neutral pH)	91.6% ENX degradation in 60 min under visible light	170
Citric acid + ethylenediamine (one-step hydrothermal)	P25 (hydrothermal)	Strong interfacial interaction between CDs and TiO_2_	Photosensitizer, electron trap, electron reservoir	˙O_2_^−^ > h^+^ > ˙OH (20 mg per L TC, 20 mg catalyst, pH 5, 70 µL CDs/TiO_2_)	100% TC degradation in 70 min under visible light	167
Lemon juice (hydrothermal)	Anatase (mixing and stirring)	van der Waals interaction & hydrogen bonding & electrostatic interaction & chemical interaction (Ti–O–C)	Electron traps, charge-transfer promoters	˙O_2_^−^ > h^+^ > ˙OH (20 mg per L TC, 200 mg per L catalyst dose, pH 8)	94.8% TC degradation in 120 min under visible light	171
Aqueous graphene quantum dots (GQDs) dispersion (not prepared)	Anatase (hydrothermal)	GQDs dispersed within Bi_2_MoO_6_ and linked to TiO_2_ (Ti–O–C) (dual Z-scheme heterojunction)	Electron mediator/photosensitizer	h^+^ > ˙O_2_^−^ > ˙OH (20 mg per L TC, and 0.4 mL GQDs BGT-2 catalyst)	78.67% TC degradation in 120 min under visible light	153
Glucose (one-step alkali-assisted ultrasonic)	Anatase (mixing and stirring)	CQDs dispersed on TiO_2_ surfaces	Electron mediator	^1^O_2_ > h^+^ > ˙O_2_^−^ > ˙OH (20 µM SMX, 50 mg per L catalyst, PH 7)	∼100% SMX degradation in 20 min under UV light	172
Lignin (dealkaline) + 1,2-diaminobenzene (hydrothermal)	Anatase (ultrasonication)	Ti–O–C chemical bonding (type II heterojunction)	Photosensitizer, transfer e^−^	h^+^ > ˙O_2_^−^ > ˙OH (10 mg per L TC, 0.5 g per L catalyst dose, pH 3)	98.6% TC degradation in 120 min under simulated sunlight	173
Polyvinylpyrrolidone	Rutile (two-step hydrothermal)	Strong interfacial interaction between TNR and In_2_S_3_, and CQDs act as an e^−^ transfer bridge (all-solid-state Z-scheme heterojunction)	Electron mediator	˙O_2_^−^ > ˙OH ≈ h^+^ (25 mg per L OFL, 0.2In/CQDs-2/TNR catalyst, pH 4.8)	92.7% OFL degradation in 120 min under simulated sunlight	117
l-Cysteine + ethylene glycol (hydrothermal)	Rutile (one-pot hydrothermal)	(Z-scheme heterojunction)	Electron acceptor and donor	˙O_2_^−^ > h^+^ > ˙OH	85.8% CIP degradation in 240 min under visible light; 44.6% under NIR light	118
Citric acid (hydrothermal)	Rutile (hydrothermal + external electric-field polarization + controlled immersion method)	TiO_2_/BTO-P core/shell interface; GQDs attached through van der Waals interactions and hydrogen bonding	Electron accumulation platform; also provides active sites for pollutant adsorption	˙O_2_^−^ ≫ h^+^ > ˙OH (20 mg per L OTC, 400 µL GQDs loading)	82.5% OTC degradation in 90 min under simulated sunlight	174

### 4.1 Photophysical excitation and charge separation

Photocatalytic degradation begins with photoinduced excitation of TiO_2_, CDs, or both, depending on the irradiation wavelength and interfacial band alignment. Under ultraviolet irradiation, TiO_2_ absorbs photons with energies equal to or greater than its bandgap, generating conduction-band electrons and valence-band holes. In this regime, CDs primarily act as electron acceptors and mediators that suppress electron–hole recombination through interfacial charge transfer.^140^

Under visible-light irradiation, degradation depends more strongly on the role of CDs as photosensitizers, because of their broad visible-light absorption, thereby extending the optical response of TiO_2_ and enabling ROS generation.^131,142,143^ Yu *et al.*^144^ demonstrated that CQDs play dual light-dependent roles in CQD/P25 composites for photocatalytic hydrogen production. Under UV-vis irradiation, TiO_2_ is directly photoexcited, while the CQDs act primarily as electron reservoirs, trapping photogenerated electrons from the conduction band of P25 and thereby suppressing electron–hole recombination and enhancing charge separation. In contrast, under visible-light irradiation (>450 nm), where P25 cannot be directly photoexcited, the π-conjugated CQDs function as photosensitizers; upon visible-light excitation, electrons are transferred from the CQDs to the conduction band of TiO_2_ through the strongly interacting Ti–O–C interface, enabling visible-light-driven photocatalytic activity.

The efficiency of interfacial electron transfer strongly depends on CD surface states, graphitization degree, heteroatom doping, and interfacial bonding configuration, as these factors collectively determine the driving force for charge migration and the density of available charge-transfer pathways.^36^ Nitrogen- and sulfur-doped CDs frequently exhibit enhanced electron-transfer capability and improved visible-light responsiveness because of modified electronic density and work-function alignment.^145,146^ Yashwanth *et al.*^80^ experimentally demonstrated that N and S doping of CQDs modifies their electronic structure by introducing localized energy states and lowering the work function, thereby facilitating photoexcitation and electron transfer. N-CQD/TiO_2_ exhibited a lower work function than S-CQD/TiO_2_ (2.89 *vs.* 3.01 eV), while N, S co-doping further reduced it to 2.53 eV and produced the highest photocatalytic activity. The co-doped system also showed enhanced visible-light absorption, suppressed electron–hole recombination, and prolonged charge-carrier lifetime, confirming a synergistic role of N and S in improving charge separation and interfacial electron transport.

### 4.2 Reactive oxygen species generation

Following charge separation, photogenerated electrons and holes participate in interfacial redox reactions that generate ROS. Conduction-band electrons reduce dissolved oxygen to form ˙O_2_^−^, whereas valence-band holes oxidize water molecules or hydroxide ions to generate ˙OH.^147,148^ In some systems, singlet oxygen (^1^O_2_) may additionally form through energy-transfer pathways from excited subsurface Ti^3+^ centers to molecular oxygen or excited-state CDs.^149^

The relative contribution of ˙OH, ˙O_2_^−^, h^+^, and ^1^O_2_ strongly depends on interfacial band alignment, oxygen adsorption behavior, surface defect states, and the redox potentials of the composite system. In many visible-light-driven CDs/TiO_2_ photocatalysts, ˙O_2_^−^ are frequently identified as the important oxidative species, because interfacial electron transfer to dissolved oxygen remains efficient, whereas valence-band oxidation may become less favorable for extensive ˙OH generation.^150^

Radical-scavenging experiments and ESR studies across multiple CDs/TiO_2_ systems consistently identify ˙O_2_^−^ and h^+^ as major oxidative species under visible-light irradiation, although ROS distributions remain strongly system-dependent.^116,151,152^

The ROS profile directly influences degradation selectivity and oxidation depth by determining which reactive species dominate the oxidation process and how strongly they interact with the target contaminants.^109,153^ Sustained ROS production is therefore essential not only for rapid parent-compound degradation but also for complete mineralization of intermediate species.

### 4.3 Molecular oxidation and degradation pathways

ROS generated at the photocatalyst surface attack antibiotic molecules adsorbed on the catalytic surface, initiating sequential oxidative transformation reactions.^98,154^ The degradation pathway strongly depends on the antibiotic molecular structure and electronic distribution. Electron-rich functional groups generally exhibit higher susceptibility to ROS attack. Consequently, ROS accessibility and interfacial electron transfer collectively determine degradation selectivity and oxidation efficiency.^20,109,155,156^

Liquid chromatography-mass spectrometry (LC-MS) analyses reported in multiple studies identify hydroxylated, dealkylated, ring-opened, and defluorinated intermediates as common transient products before mineralization.^157,158^ In tetracycline systems, degradation frequently occurs by oxidation of electron-rich functional groups, with demethylation, hydroxylation, and cleavage of the polycyclic framework.^159,160^ Fluoroquinolone degradation commonly involves piperazine-ring scission, decarboxylation, oxidation, and defluorination prior to destruction of the quinolone core structure.^155,161^ β-Lactam antibiotics generally undergo oxidative opening of the strained β-lactam ring.^162,163^ Whereas sulfonamides are primarily degraded through cleavage of S–N, C–N, and S–C bonds and hydroxylation reactions between sulfur and the benzene ring.^164^

Although disappearance of the parent antibiotic may occur rapidly, complete mineralization generally proceeds more slowly because partially oxidized intermediates can accumulate during multistep degradation.^165^ Total organic carbon (TOC) analyses consistently demonstrate that mineralization efficiencies lag behind apparent degradation rates, emphasizing the importance of sustained ROS generation and efficient interfacial charge separation for deep oxidation.

### 4.4 Kinetic behavior and interfacial control

The overall photocatalytic degradation process is influenced by surface reaction kinetics and adsorption equilibrium. Antibiotic degradation over CDs/TiO_2_ nanocomposites commonly follows an apparent pseudo-first-order kinetic model, while the Langmuir and Freundlich models are used to describe adsorption isotherms.^165,166^ Qurtulen *et al.*^139^ reported that ciprofloxacin (CIP) degradation over CQDs/TiO_2_ followed pseudo-first-order kinetics, with the CQDs/TiO_2_ composite exhibiting superior kinetic performance compared with bare TiO_2_ and CQDs. Equilibrium adsorption was well described by both Freundlich and Langmuir isotherms, with the Freundlich model showing a slightly better fit (*R*^2^ = 0.95) than the Langmuir model (*R*^2^ = 0.93). The Langmuir model yielded a maximum adsorption capacity of 406.5 mg g^−1^, whereas the Freundlich fitting indicated heterogeneous adsorption sites on the composite surface. The composite also exhibited a high BET surface area of 197 m^2^ g^−1^ and achieved 97.31% CIP degradation within 50 min. Collectively, these results suggest that favorable surface adsorption, together with enhanced photocatalytic activity, contributes to the efficient removal of CIP by the CQDs/TiO_2_ nanocomposite.

Kinetic studies reported by Xie *et al.*^167^ demonstrated that optimized CD loading substantially increases apparent pseudo-first-order rate constants relative to pristine TiO_2_ because of improved interfacial charge transfer and suppressed recombination. However, excessive carbon dosage can reflect and scatter the incident light, leading to a decrease in light transmittance and an increase in charge recombination, which results in decreased degradation ability.

Therefore, efficient photocatalytic degradation requires balanced heterointerface construction capable of simultaneously maximizing spectral harvesting, carrier separation, and ROS generation. The overall degradation performance of CDs/TiO_2_ nanocomposites thus emerges from coordinated coupling between interfacial electronic structure and surface reaction kinetics under environmentally relevant operating conditions.

## 5 CDs/TiO_2_ nanocomposites for major antibiotic classes

CDs/TiO_2_ nanocomposites exhibit antibiotic-class-dependent photocatalytic behavior because degradation efficiency is governed by the interplay between molecular electronic structure, adsorption affinity, ionization state, and reactive oxygen species (ROS) selectivity. Variations in aromatic conjugation, heteroatom distribution, electron-donating substituents, and bond dissociation energies strongly influence adsorption equilibrium, preferred ROS attack sites, and mineralization pathways. Consequently, heterointerface engineering within CDs/TiO_2_ systems not only regulates charge separation and ROS generation but also determines degradation selectivity toward different classes of antibiotic pollutants.

### 5.1 Tetracyclines

Tetracyclines contain a polycyclic naphthacene framework with multiple hydroxyl, carbonyl, and dimethylamino substituents that provide abundant electron-rich reactive sites.^156^ Their extended π-conjugated structure promotes strong π–π interactions with graphitic domains of CDs, thereby ensuring a rapid and effective photocatalytic process.^175,176^ Xie *et al.*^174^ reported that, in the TiO_2_/BTO-P/GQDs system for OTC degradation, the π-conjugated structure of GQDs serves as both an electron-accumulation platform and a chemically active surface. The accumulated electrons react with O_2_ to generate ˙O_2_^−^ radicals, while the GQD surface facilitates pollutant adsorption and activation, thereby synergistically enhancing photocatalytic degradation.

Consequently, tetracyclines are generally among the most efficiently degraded antibiotic classes in CDs/TiO_2_ photocatalytic systems.

As illustrated in Fig. 12a and b, Liu *et al.*^177^ proposed a visible-light-driven degradation mechanism for tetracycline over CQD–OTNs photocatalysts in which h^+^ and ˙O_2_^−^ played important roles. In this system, CQDs primarily function as electron acceptors and interfacial charge-transfer mediators that improve carrier separation and suppress recombination. The degradation sequence involves oxidation of electron-rich functional groups, with hydroxylation, and progressive cleavage of the polycyclic framework before mineralization.

**Fig. 12:**
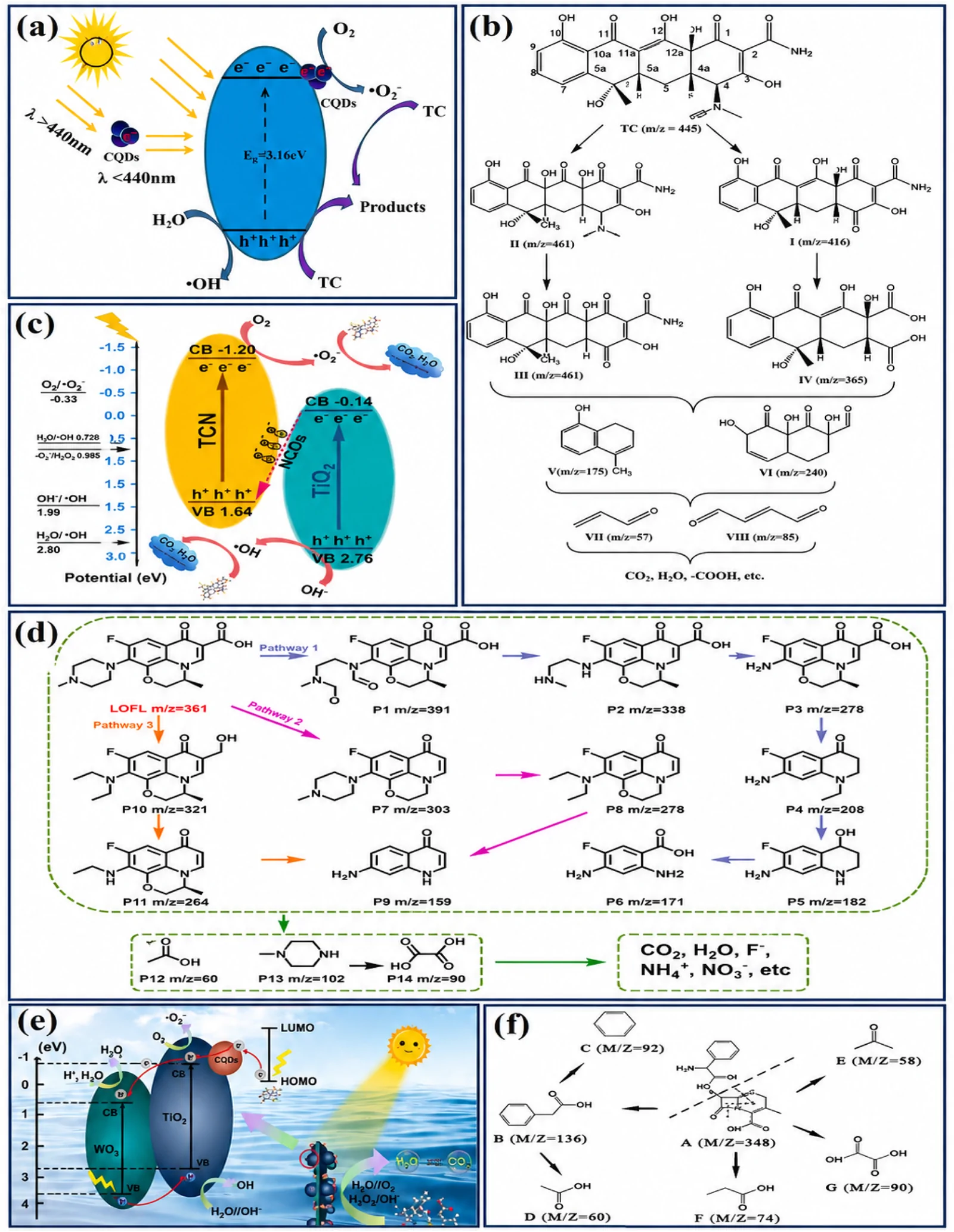
(a) Proposed photocatalytic mechanism for tetracycline degradation over CQD–OTNs; (b) tetracycline degradation pathway under visible-light irradiation. Reproduced with permission from ref. 177 copyright 2020, Elsevier. (c) Proposed Z-scheme charge-transfer mechanism for levofloxacin degradation in TiO_2_/NCDs/TCN systems; (d) representative levofloxacin degradation pathway. Reproduced with permission from ref. 169 copyright, 2025, Elsevier. (e) Photocatalytic degradation mechanism of cephalexin over TiO_2_/WO_3_/CQDs heterojunctions; (f) proposed cephalexin degradation pathway under irradiation conditions. Reproduced with permission from ref. 142 copyright, 2021, Elsevier.

Karaca *et al.*^79^ further demonstrated that nitrogen-doped CDs/TiO_2_ systems significantly accelerate tetracycline degradation because nitrogen incorporation improves charge delocalization and decreases the work function, also providing access to tunable electronic and optical properties. Similarly, Li *et al.*^159^ reported that oxygen-vacancy-rich TiO_2_ stabilized by nitrogen-doped CDs enhances ROS formation and promotes deeper oxidation of tetracycline intermediates to mineralization.

Liquid chromatography-mass spectrometry (LC-MS) analyses consistently identify hydroxylated intermediates, demethylated products, and ring-opened species before mineralization.^153,167^ Although parent-compound removal frequently occurs rapidly, complete mineralization proceeds more slowly because partially oxidized intermediates may accumulate during multistep degradation.^116^ Systems exhibiting strong Ti–O–C interfacial coupling and optimized CD loading generally achieve higher total organic carbon (TOC) removal efficiencies, indicating sustained ROS generation and improved deep oxidation capability.^109^

### 5.2 Fluoroquinolones

Fluoroquinolones are characterized by a carboxylic acid functional group and a quinolone aromatic core containing fluorine substituents and piperazinyl groups.^178^ The electron-withdrawing fluorine atom reduces electron density within the aromatic framework, thereby increasing resistance toward electrophilic ROS attack and slowing complete mineralization. Consequently, compared with tetracyclines, degradation efficiency in CDs/TiO_2_ systems depends more strongly on sustained ROS generation and efficient interfacial charge transfer.

Under visible-light irradiation, CDs can function as UCPL, photosensitizers and electron-transfer mediators that promote superoxide-radical generation through electron injection into the TiO_2_ conduction band.^99,152^ As illustrated in Fig. 12c and d, Guo *et al.*^169^ proposed a Z-scheme photocatalytic mechanism for levofloxacin degradation over TiO_2_/NCDs/TCN heterojunctions, where incorporation of nitrogen-doped CDs substantially improves interfacial charge separation and directional electron migration; NCDs serve as an excellent interfacial electron-transport bridge between TiO_2_ and TCN. The proposed degradation pathway involves piperazine-ring cleavage together with decarboxylation, oxidation, demethylation, and defluorination reactions, followed by ring opening and progressive destruction of the quinolone framework into lower-molecular-weight products.^169^ Su *et al.*^170^ further reported that optimized CD loading in CDs/TiO_2_/g-C_3_N_4_ systems enhances Z-scheme charge transfer and improves enrofloxacin degradation.

Because the aromatic heterocycle contributes significantly to fluoroquinolone stability, efficient mineralization requires continuous ROS generation and prolonged carrier lifetime to prevent accumulation of partially oxidized intermediates.

### 5.3 β-Lactams

β-Lactam antibiotics contain a strained four-membered lactam ring that is highly susceptible to oxidative cleavage.^162,179^ However, unlike tetracyclines and fluoroquinolones, many β-lactams possess relatively limited aromatic conjugation, reducing π–π interactions with graphitic CD domains. Consequently, adsorption depends more strongly on electrostatic attraction by surface functional groups on CDs, depending on the pH.^40^ Viscusi *et al.*^180^ used Ni@TiO_2_/NCD (4%) for photocatalytic degradation of ceftriaxone, achieving 70% removal and 65% TOC reduction after 180 min of simulated solar-light irradiation. During the preceding dark adsorption stage, at the spontaneous solution pH of approximately 6.5, the Ni@TiO_2_/NCD surface was negatively charged because the pH exceeded its PZC (5.2). Ceftriaxone, which predominantly exists in an anionic or zwitterionic form at this pH, exhibited relatively low adsorption because of electrostatic repulsion with the negatively charged catalyst surface. These findings highlight the importance of electrostatic interactions in governing the adsorption–degradation interplay, and also explain the higher initial removal observed for vancomycin compared to ceftriaxone.

As illustrated in Fig. 12e and f, Sun *et al.*^142^ proposed a photocatalytic degradation mechanism for cephalexin over TiO_2_/WO_3_/CQDs heterojunctions under simulated solar irradiation. In this ternary heterostructure, CQDs function as electron reservoirs and photosensitizers that facilitate interfacial charge separation and ROS generation. Photocatalytic degradation by oxidative opening of the β-lactam ring, with fragmentation and progressive oxidation to lower-molecular-weight intermediates before mineralization. Chen *et al.*^181^ additionally demonstrated that CQD-modified TiO_2_ nanocomposites improve cefradine degradation through enhanced interfacial electron transfer and increased hydroxyl-radical generation.

β-Lactam antibiotics can undergo rapid initial degradation through pathways that include β-lactam-ring opening. However, mineralization may proceed more slowly because of the formation and persistence of organic intermediates that require further oxidation to achieve complete conversion into inorganic end products.^142,182^ Therefore, sustained ROS generation and stable interfacial charge separation remain essential for efficient deep oxidation.

### 5.4 Sulfonamides

Sulfonamides contain sulfonyl-linked aromatic amine structures that exhibit amphoteric behavior and environmental persistence.^183^ Sulfonamides are primarily degraded through cleavage of S–N, C–N, S–C bonds, and amino-group oxidation and aromatic hydroxylation reactions.^162,184^

Wei *et al.*^172^ reported that CQDs@LM-TiO_2_ systems used for photocatalytic activation of periodate under ultraviolet light resulted in dominant singlet oxygen (_1_O^2^) generation during sulfamethoxazole degradation. Multiple degradation pathways involve S–N bond cleavage, hydroxylation, amino-group oxidation, and subsequent ring-opening reactions. Also, Ullah *et al.*^185^ investigated lignin-derived carbon quantum dot–sensitized TiO_2_ (L–CQDs/TiO_2_) for the visible-light photocatalytic degradation of sulfamethoxazole (SMX). The optimized 3 wt% L–CQDs/TiO_2_ composite exhibited a markedly enhanced photocatalytic performance, achieving 98.6% SMX degradation within 90 min, whereas pristine TiO_2_ showed only 2% degradation under the same conditions.

Sulfonamide degradation is particularly sensitive to environmental matrix composition. Solution pH, inorganic ions, and natural organic matter strongly influence ROS availability, light absorption, and degradation selectivity. Sulfate, nitrate, and bicarbonate ions may scavenge reactive radicals or alter interfacial charge-transfer pathways, thereby reducing apparent degradation efficiency.^87,141^

### 5.5 Comparative performance benchmarking

Comparative evaluation across antibiotic classes demonstrates that CDs/TiO_2_ nanocomposites consistently outperform pristine TiO_2_ because of enhanced spectral harvesting, improved charge separation, and stronger degradation capability.^161,186^ Tetracyclines generally exhibit the highest degradation rates because of multiple electron-rich reactive sites and strong π–π interactions. Fluoroquinolones display intermediate degradation behavior governed by electron-transfer efficiency and defluorination kinetics, whereas β-lactams often undergo rapid initial ring opening followed by slower mineralization. Sulfonamides can undergo degradation through multiple pathways.

Lu *et al.*^155^ employed a ternary Bi_2_MoO_6_/GQDs/TiO_2_ photocatalyst for the degradation of ciprofloxacin (CIP), amoxicillin (AMX), and tetracycline hydrochloride (TC-HCl), achieving degradation efficiencies of 90.21%, 78.98%, and 92.49%, respectively. As shown in Fig. 13c and d, the time-dependent photodegradation profiles and pseudo-first-order kinetic constants. Although many CDs/TiO_2_ photocatalysts exhibit apparent degradation-rate constants substantially higher than pristine TiO_2_, complete mineralization remains more challenging than parent-compound removal, as evidenced by lower TOC removal efficiencies. This observation highlights the importance of sustained ROS generation and stable interfacial charge-transfer pathways for deep oxidation.

**Fig. 13:**
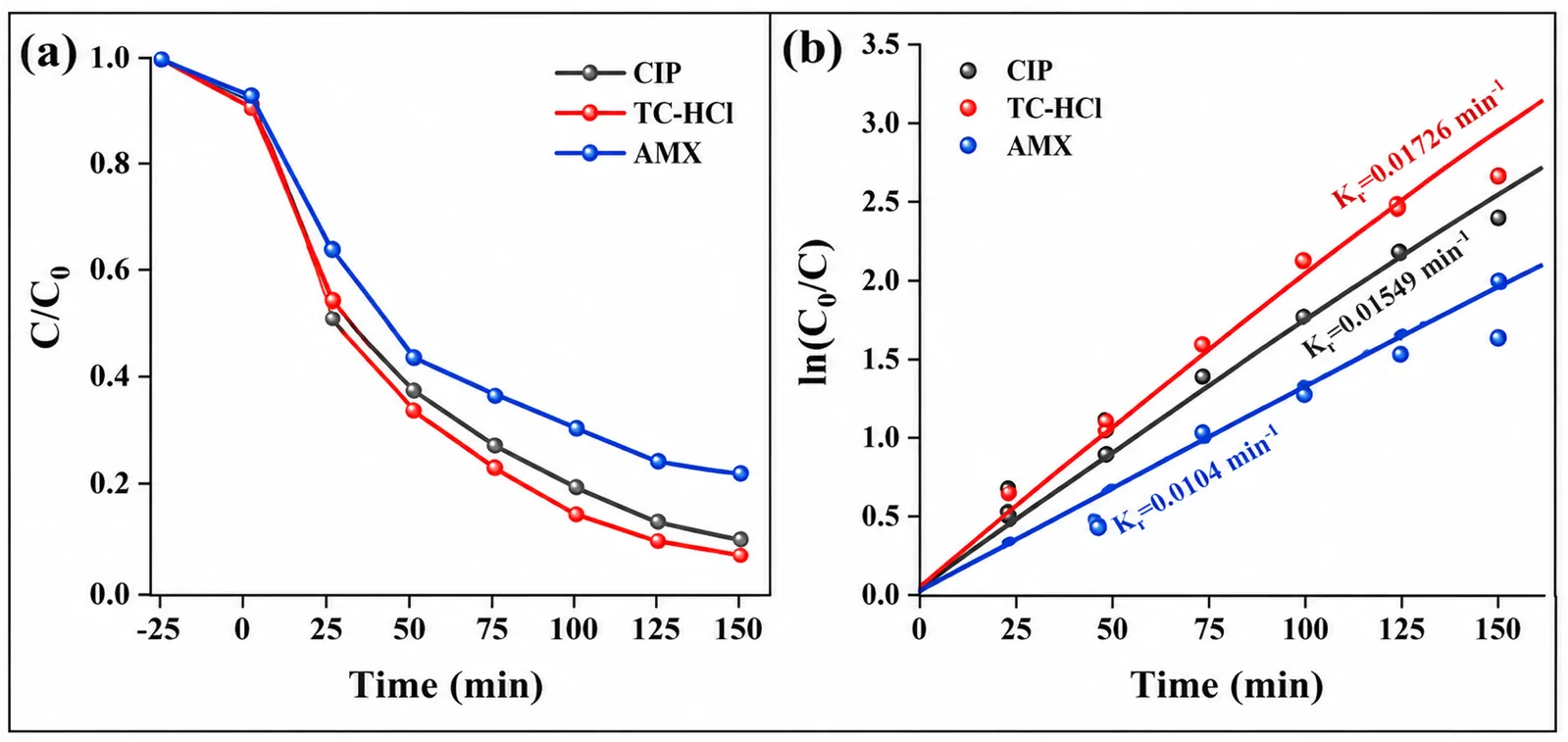
Comparative photocatalytic performance of representative Bi_2_MoO_6_/CDs/TiO_2_ nanocomposites toward major antibiotic classes. (a) Time-dependent degradation profiles under irradiation conditions; (b) apparent pseudo-first-order kinetic constants for tetracyclines, fluoroquinolones, β-lactams. Adapted with permission from ref. 155 copyright 2022, Elsevier.

Carbon dots (CDs) have emerged as a versatile class of carbon-based nanomaterials with considerable potential in photocatalytic applications, offering several advantages, including tunable optical properties, efficient visible-light sensitization, and versatile surface functionalization capability.^187,188^ However, direct benchmarking across studies remains difficult because of variations in irradiation conditions, reactor configurations, catalyst dosage, pollutant concentration, and analytical methods. Consequently, increasing emphasis has been placed on standardized reporting of pseudo-first-order rate constants normalized to catalyst mass, photon flux, and mineralization efficiency.

A quantitative comparison of representative CDs/TiO_2_ photocatalysts reported for different antibiotic classes is summarized in Table 2, highlighting variations in degradation kinetics, mineralization efficiency, irradiation conditions, and catalyst composition.

**Table 2:** Comparative photocatalytic performance of CDs/TiO_2_ nanocomposites for antibiotic degradation

Antibiotic	Catalyst system	Catalytic dosage (g L^−1^)	Initial concentration (mg L^−1^)	Light source	*k*_app_ (min^−1^)	Degradation (%) (time)	TOC removal (%) (time)	Ref.
Tetracycline	RCDs/TiO_2_	0.5	10	300 W xenon lamp (simulated sunlight)	0.0493	95 (60 min)	57.5 (60 min)	189
Tetracycline	PTh/GQDs/TiO_2_	—	20	300 W xenon lamp (visible-light irradiation)	0.0087	80.58 (150 min)	29.1 (150 min)	166
Tetracycline	TiO_2_/CQDs	0.5	20	300 W xenon lamp (visible-light irradiation)	0.1345	98 (30 min)	∼20 (30 min)	116
Tetracycline	CDs/TiO_2_	0.4	20	Xenon lamp (visible-light irradiation)	0.0534	100 (70 min)	50.1 (70 min)	167
Tetracycline	CQDs/TiO_2_	0.2	20	Xenon lamp (visible-light irradiation)	0.01949	91.5 (120 min)	42 (120 min)	171
Tetracycline	CQD–OTNs	0.5	15	500 W xenon lamp (visible-light irradiation)	0.0335	94.1 (120 min)	26.52 (120 min)	177
Tetracycline	VP-CQDs/TiO_2_	1.5	10	300 W mercury lamp (simulated solar light)	0.0216	97.34 (120 min)	71.99 (120 min)	109
Tetracycline	CQDs@TiO_2_	0.2	10	Xenon lamp (visible-light irradiation)	—	99.5 (120 min)	44.8 (120 min)	190
Tetracycline	TiO_2_/N-CDs	0.6	15	Sunlight	0.030	96 (90 min)	85 (90 min)	143
Levofloxacin	TiO_2_/CDs	0.25	10	Sunlight	0.01826	∼99 (90 min)	53.4 (90 min)	98
Levofloxacin	Bi_2_WO_6_/CDs/TiO_2_	0.075	10	Sunlight	0.0466	99 (90 min)	64 (90 min)	121
Enrofloxacin	Sr-doped TiO_2_/CDs	0.05	10	250 W gold halide lamp (simulated visible-light irradiation)	0.0118	84.7 (180 min)	61 (180 min)	161
Enrofloxacin	CDs–TiO_2_/SiC_f_	0.07	10	250 W metal halide lamp (simulated visible light)	—	83.1 (180 min)	51.3 (180 min)	191
Cephalexin	TiO_2_/WO_3_/CQDs	0.5	10	300 W xenon lamp (simulated solar light)	0.0460	94 (60 min)	92.4 (4 h)	142
Tetracycline	NPCDs/(001)TiO_2_/Ti_3_C_2_T_*x*_	0.5	20	480 W xenon lamp (simulated solar light)	0.01124	93.4 (150 min)	54.65 (150 min)	192
Ceftiofur sodium			25		0.00738	75.5 (150 min)	43.53 (150 min)	
Sulfamethoxazole	Cu–TiO_2_/CQDs	0.8	20	Xenon lamp (visible-light irradiation)	0.0541	100% (60 min)	81 (60 min)	141

## 6 Factors influencing photocatalytic performance

Although interfacial engineering determines the intrinsic catalytic potential of CDs/TiO_2_ nanocomposites, the observed photocatalytic performance ultimately depends on the coupled interaction between material architecture, operational parameters, environmental matrix composition, and antibiotic molecular properties. Consequently, photocatalytic efficiency emerges from a multidimensional balance involving charge-transfer dynamics, adsorption equilibrium, photon utilization, and reactive oxygen species (ROS) accessibility under realistic operating conditions. The major parameters governing photocatalytic antibiotic degradation in CDs/TiO_2_ systems are summarized in Fig. 14a–d.

**Fig. 14:**
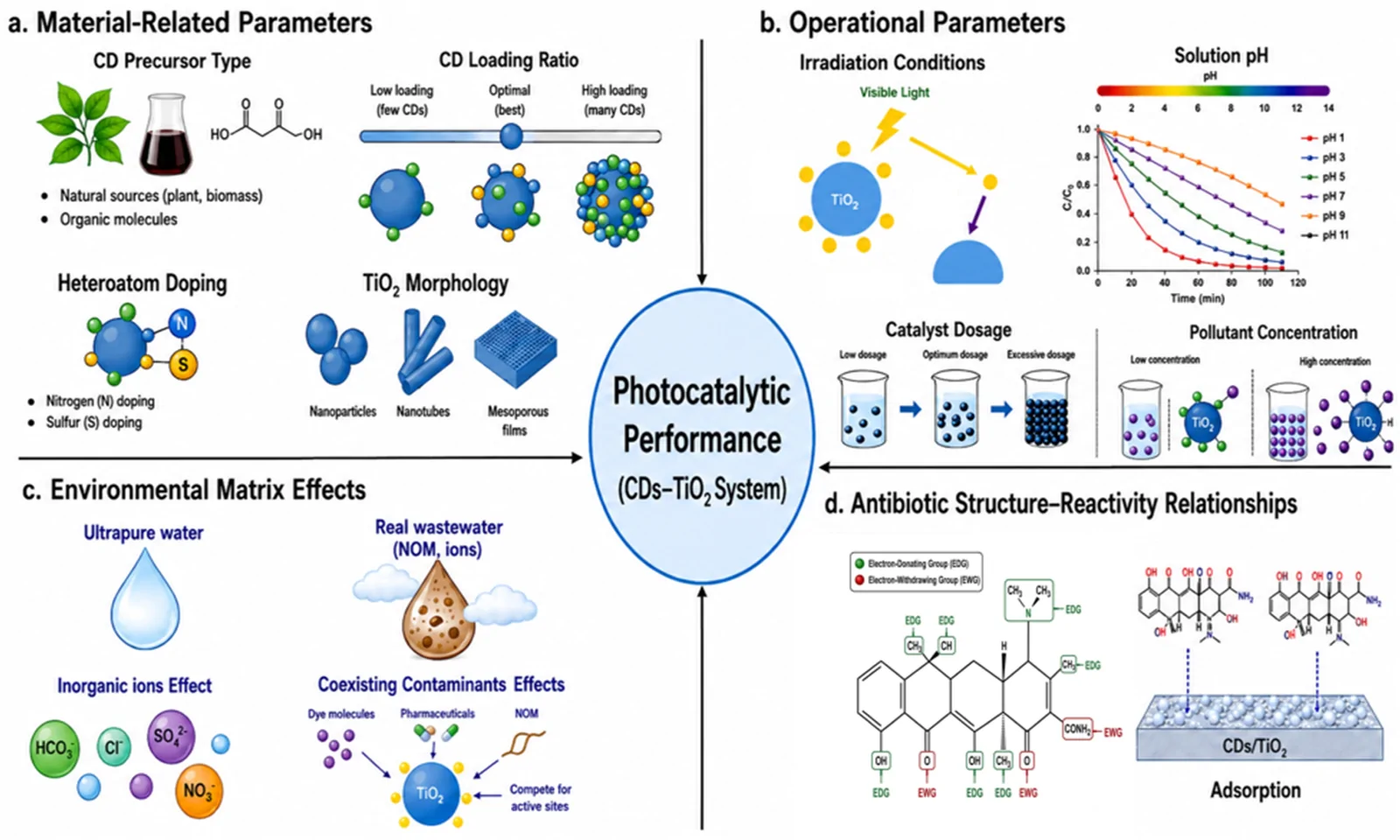
Schematic overview of the multidimensional factors governing photocatalytic antibiotic degradation in CDs/TiO_2_ nanocomposites, encompassing material-related parameters, operational variables, environmental matrix effects, and antibiotic structure–reactivity relationships. (a) CD precursor type, heteroatom doping, loading ratio, and TiO_2_ morphology directly influence band alignment, defect-state distribution, interfacial charge transfer, and ROS generation efficiency. (b) Light irradiation, PH value, catalyst dosage, and pollutant concentration strongly regulate photocatalytic degradation. (c) Environmental matrix effects, including radical scavenging and competitive adsorption. (d) Structure–reactivity relationships linking antibiotic molecular properties with ROS susceptibility and degradation.

### 6.1 Material-related parameters

The structural and electronic properties of CDs/TiO_2_ nanocomposites are highly sensitive to synthesis conditions and compositional design.^110,136,193^ As illustrated in Fig. 14a, CD precursor type, heteroatom doping, loading ratio, and TiO_2_ morphology directly influence band alignment, defect-state distribution, interfacial charge transfer, and ROS generation efficiency.

Heteroatom doping, particularly with nitrogen and sulfur atoms, can substantially modify the electronic structure of CDs, alter work-function alignment, and improve visible-light responsiveness.^146,168^ Compared with undoped CDs, nitrogen-doped CDs frequently exhibit enhanced photocatalytic performance in TiO_2_ based composed because of improved interfacial electron transfer and reducing work-function induced by nitrogen doping.^194^

The CD loading ratio also plays a critical role in determining photocatalytic activity. At insufficient loading levels, limited interfacial contact restricts sensitization and charge-carrier mediation. In contrast, excessive carbon coverage may shield TiO_2_ active sites, reduce photon penetration, and introduce additional recombination centers.^195,196^ Shen *et al.*^197^ demonstrated the existence of an optimum CD concentration beyond which the apparent degradation rate progressively declines because of increased recombination probability and surface-blockage effects.

In addition to compositional tuning, the morphology and crystal phase of TiO_2_ strongly influence surface accessibility, mass transport, and optical utilization efficiency. Anatase generally exhibits favorable band-edge positions for ROS generation,^108,198^ whereas mixed-phase systems may improve carrier mobility and interfacial charge migration.^140^ Nanostructured architectures, including nanotubes, mesoporous films, and hierarchical assemblies, provide increased surface area and enhanced adsorption capability, although strong interfacial coupling with CDs remains essential for maintaining electronic synergy.^199–201^

### 6.2 Operational parameters

Operational conditions strongly regulate photocatalytic degradation behavior, as illustrated in Fig. 14b. The spectral distribution and intensity of the irradiation source determine the dominant excitation pathway within CDs/TiO_2_ systems. Under ultraviolet irradiation, photocatalytic activity is primarily governed by direct TiO_2_ excitation, whereas under visible-light or solar irradiation, degradation efficiency depends more strongly on CD sensitization and interfacial electron transfer.^140,142^

Light intensity additionally influences degradation efficiency because increased photon flux enhances electron–hole generation and ROS formation.^172,202^ However, excessive irradiation intensity may accelerate carrier recombination. Therefore, optimization of irradiation conditions is necessary to balance ROS production with charge-carrier utilization efficiency.

Solution pH strongly affects catalyst surface charge and antibiotic speciation. Because both the photocatalyst surface charge and antibiotic ionization state are pH-dependent, electrostatic attraction or repulsion can substantially alter adsorption and degradation efficiency.^139,165^

Catalyst dosage also affects photocatalytic performance by regulating the number of available active sites and photon absorption efficiency. Increasing catalyst concentration generally improves degradation rates up to an optimum threshold, beyond which excessive particle aggregation and light scattering reduce effective photon penetration.^109,191,203^ Similarly, elevated pollutant concentrations progressively occupy active sites and limit light penetration to the catalyst, thereby decreasing apparent pseudo-first-order degradation constants.^181^

### 6.3 Environmental matrix effects

Photocatalytic efficiencies measured under laboratory conditions frequently overestimate practical environmental performance because most experiments are conducted using simplified aqueous matrices.^151,155^ As illustrated in Fig. 14c, natural organic matter (NOM) influences photocatalytic degradation through multiple competing mechanisms, including photon attenuation, competitive adsorption, and ROS scavenging, collectively reducing oxidation efficiency.

Inorganic ions additionally influence photocatalytic pathways through radical scavenging and interfacial electrostatic interactions. Bicarbonate ions may convert hydroxyl radicals into less reactive carbonate radicals, whereas chloride ions can participate in secondary reactive chlorine species formation or adsorb on the catalytic surface under certain conditions.^117,151,204,205^ Dissolved ions may also alter electrical double-layer interactions and interfacial charge-transfer pathways, thereby affecting adsorption behavior and ROS accessibility.

Coexisting contaminants, including dyes, pharmaceuticals, and natural organic matter, may further compete for active sites and consume photogenerated charge carriers or reactive radicals.^87,109^ Consequently, degradation efficiencies obtained in ultrapure laboratory systems should be interpreted cautiously when predicting large-scale environmental performance.

### 6.4 Antibiotic structure–reactivity relationships

The intrinsic molecular properties of antibiotics strongly influence photocatalytic degradation behavior and adsorption equilibrium, as summarized in Fig. 14d. Functional groups, including hydroxyls, tertiary amines, aromatic rings, and heterocyclic nitrogen atoms, frequently serve as preferential ROS attack sites.^151,204,206^ Electron-donating substituents generally enhance susceptibility toward ROS oxidative attack, whereas electron-withdrawing groups stabilize certain portions and direct ROS attack toward other reactive centers.^20^

Extended π-conjugated structures facilitate adsorption through π–π interactions with graphitic CD domains, thereby improving interfacial oxidation probability and degradation kinetics. In contrast, weakly conjugated antibiotics depend more strongly on hydrogen bonding and electrostatic interactions for surface adsorption.^40,92,141,207^

Density functional theory (DFT) calculations further support experimental observations by identifying high-electron-density regions and the highest occupied molecular orbital (HOMO) localization as preferential electrophilic radical attack sites.^171,172^ These computational insights provide valuable guidance for tailoring interfacial properties toward selective degradation of structurally distinct antibiotic classes.

Collectively, these factors demonstrate that photocatalytic efficiency in CDs/TiO_2_ systems emerges from the coupled interaction between catalyst architecture, operational conditions, environmental matrix composition, and antibiotic molecular structure. Therefore, effective optimization requires coordinated multi-parameter tuning rather than isolated modification of a single variable. This systems-level perspective is essential for translating laboratory-scale photocatalytic activity into reliable environmental remediation performance.

## 7 Environmental remediation applications

Translation of CDs/TiO_2_ photocatalysts from laboratory-scale mechanistic studies toward practical environmental remediation requires coordinated optimization of catalyst architecture, reactor engineering, photon utilization, hydrodynamic behavior, and environmental compatibility. Although heterointerface engineering substantially improves intrinsic photocatalytic activity, practical treatment performance additionally depends on operational robustness, catalyst stability, and efficient mass-transfer processes under realistic environmental conditions.

### 7.1 Laboratory-scale validation and kinetic evaluation

Most photocatalytic investigations of CDs/TiO_2_ nanocomposites are conducted in batch photoreactors under controlled irradiation conditions to evaluate degradation kinetics, mineralization efficiency, and ROS contributions.^98,208^ These systems commonly employ simulated solar irradiation or visible-light sources together with controlled catalyst dosage and antibiotic concentration.^167^ Compared with pristine TiO_2_, CDs/TiO_2_ photocatalysts consistently demonstrate enhanced degradation efficiency because of improved spectral harvesting and interfacial charge separation.^186,197^

Despite these promising results, laboratory-scale studies frequently rely on simplified aqueous matrices and idealized irradiation environments that do not fully represent realistic wastewater conditions. Variations in photon flux, reactor geometry, and hydrodynamic behavior further complicate direct comparison among studies.^209^ Consequently, laboratory-scale experiments should primarily be interpreted as mechanistic validation platforms rather than direct predictors of full-scale environmental performance.

### 7.2 Reactor engineering and scale-up strategies

Practical implementation of CDs/TiO_2_ photocatalysts requires a transition from laboratory slurry systems toward engineered reactor configurations capable of sustaining efficient photon utilization, catalyst stability, and operational scalability.

Immobilized-film systems provide high surface area, simplify catalyst recovery and reuse, and reduce the need for post-treatment separation.^210,211^ Tian *et al.*^212^ used a dynamic piezo-photocatalytic film reactor as illustrated in Fig. 15a. The PVDF/TiO_2_/CQDs film was fixed inside a stainless-steel cell with a quartz glass cover, so visible light could reach the film. Solution was pumped over the film surface. The flowing solution created pressure on the PVDF film, producing a piezoelectric field, while light activated TiO_2_/CQDs. Together, these effects improved adsorption, charge separation, and reactive radical formation, leading to faster dye and tetracycline degradation. The reactor removed 95.26% RhB and 91.82% tetracycline in 100 min.

**Fig. 15:**
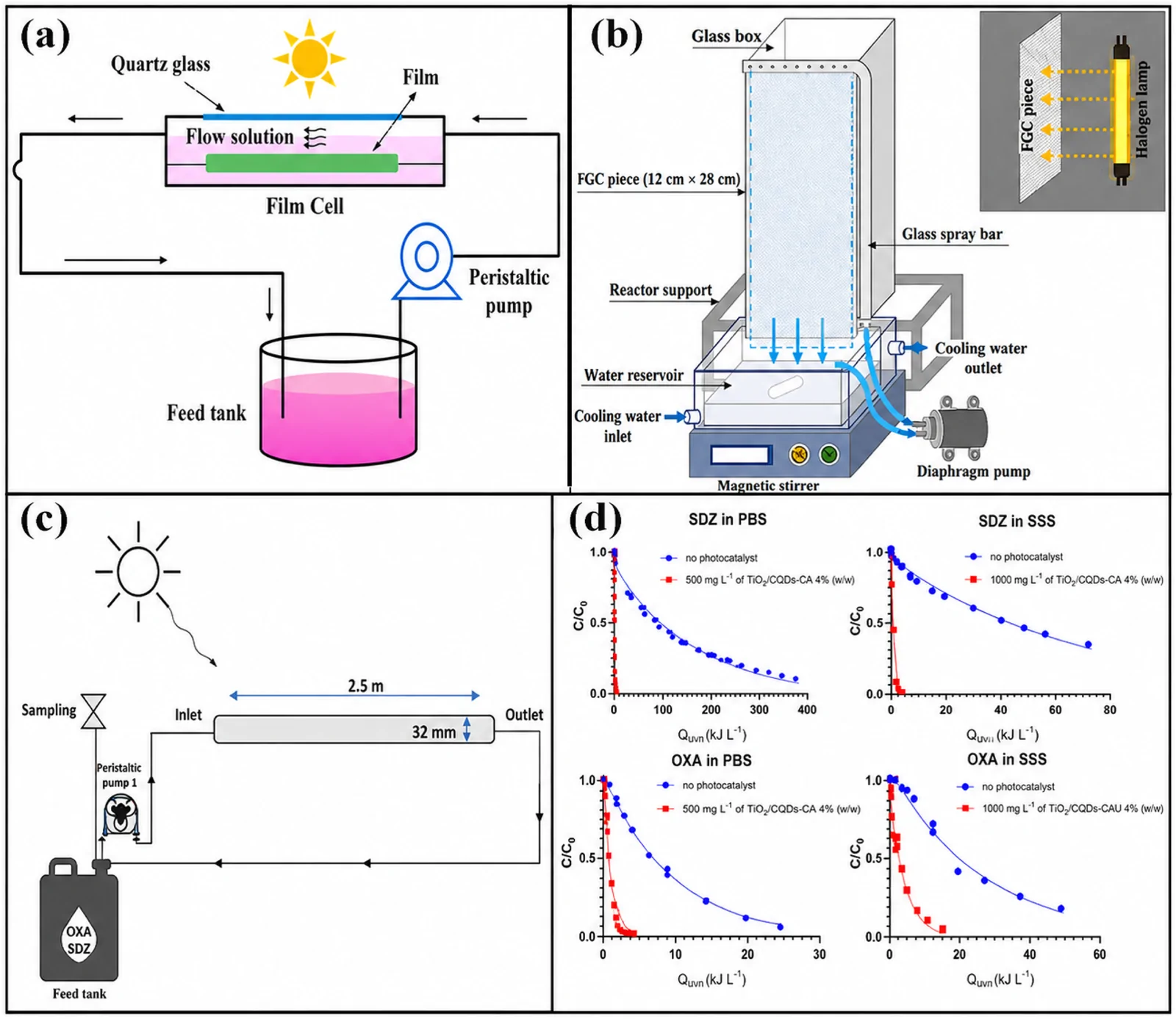
Schematic illustrations of reactor configurations and photocatalytic performance for remediation applications of CDs/TiO_2_ nanocomposites. (a) Dynamic piezo-photocatalytic film reactor employing a PVDF/TiO_2_/CQDs film fixed inside a stainless-steel cell with a quartz glass cover. Adapted with permission from ref. 212 copyright 2024, Elsevier. (b) Recirculating reactor with 5CQDs/TiO_2_-coated fiberglass cloth as the active photocatalytic surface, where the pharmaceutical solution is pumped to a glass spray bar and continuously circulated over the vertically positioned catalyst under visible-light irradiation. Adapted with permission from ref. 213 copyright 2023, Elsevier. (c) Solar-driven tubular flow photoreactor enabling continuous recirculation of contaminated water under natural solar irradiation; (d) degradation performance of sulfadiazine and oxolinic acid in phosphate-buffered solution and sea-salt solution under solar irradiation conditions, evaluated as a function of accumulated UV energy (QUV). Adapted with permission from ref. 215 copyright 2024, Elsevier.

Muangmora *et al.*^213^ used a recirculating reactor. As illustrated in Fig. 15b, the 5CQDs/TiO_2_-coated fiberglass cloth acted as the active photocatalytic surface for pharmaceutical degradation. The pharmaceutical solution was pumped to a glass spray bar and continuously circulated over the vertically positioned catalyst under visible-light irradiation, resulting in degradation efficiencies of 82% caffeine, 88% carbamazepine, and 84% ibuprofen after 9 h.

Solar-driven flow photoreactors have emerged as particularly attractive systems because they combine continuous operation with improved photon-transfer efficiency and reduced external energy consumption.^214^ Silva *et al.*^215^ demonstrated that coupling TiO_2_ with carbon quantum dots significantly enhanced solar-driven degradation of sulfadiazine and oxolinic acid in simulated freshwater and brackish aquaculture effluents. As illustrated in Fig. 15c and d, the tubular solar-flow reactor enabled continuous recirculation of contaminated water under natural solar irradiation, while degradation efficiency was evaluated as a function of accumulated UV energy (QUV), providing a useful basis for reactor comparison and scale-up analysis.

Reactor hydrodynamics, residence-time distribution, and light attenuation collectively govern photocatalytic performance under continuous-flow operation.^209,216^ Therefore, large-scale remediation efficiency depends not only on intrinsic catalyst activity but also on optimized reactor architecture and photon-management strategies.

### 7.3 Hybrid treatment integration

Photocatalysis is increasingly investigated as part of integrated treatment systems rather than as an isolated remediation technology.^217^

In membrane–photocatalyst hybrid systems, incorporation of photocatalytic materials can improve membrane antifouling performance while simultaneously enhancing photocatalytic degradation of organic pollutants.^218^ Vahedi *et al.*^138^ reported a visible-light-responsive g-C_3_N_4_/TiO_2_/CQD/EPVC photocatalytic membrane exhibiting enhanced charge separation, improved spectral utilization, and effective self-cleaning behavior. The synergistic integration of g-C_3_N_4_, TiO_2_, and CQDs intensified oxidative degradation of retained pollutants while improving membrane flux recovery Fig. 16a.

**Fig. 16:**
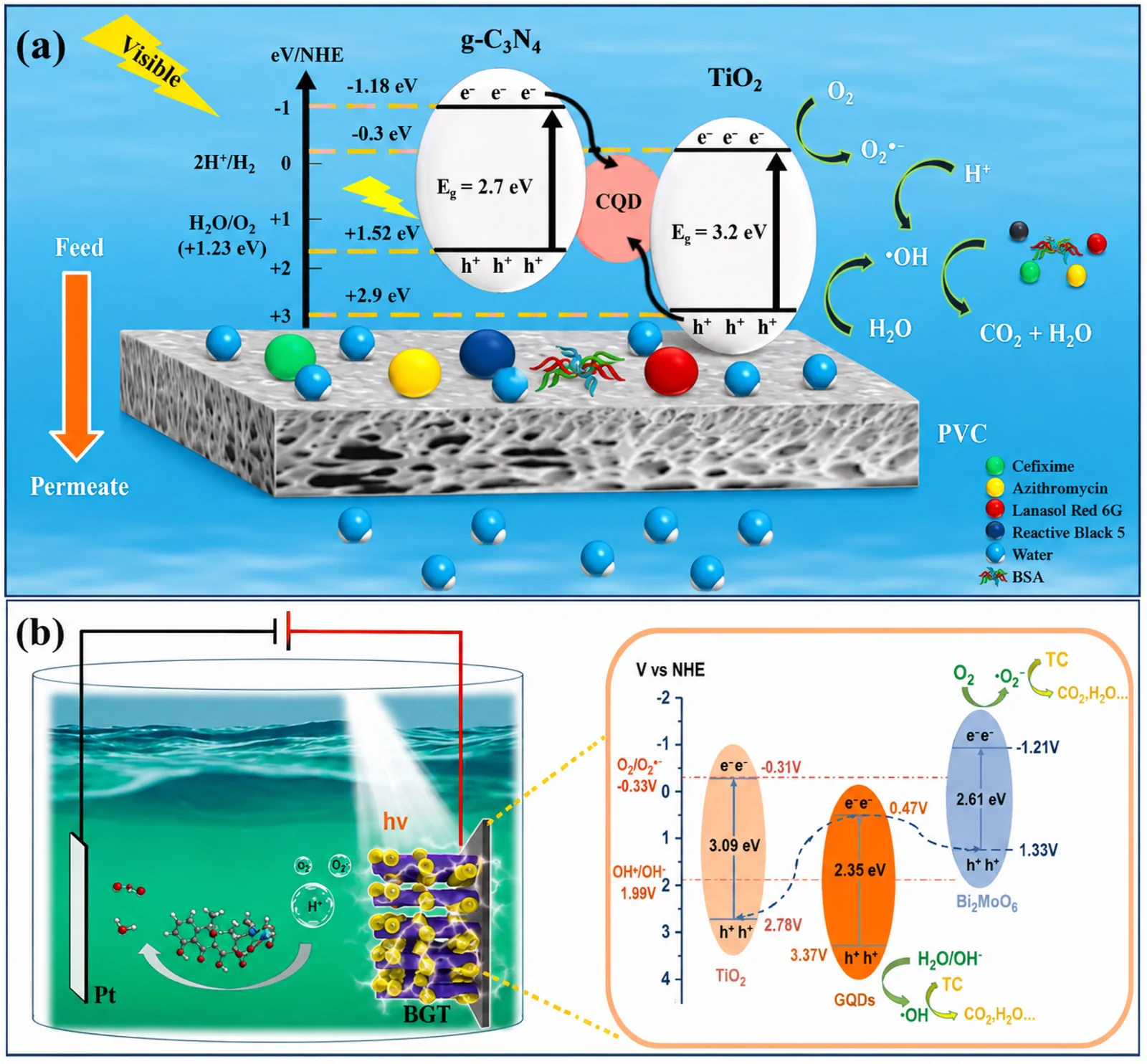
Schematic illustrations of hybrid treatment systems integrating CDs/TiO_2_ photocatalysts with remediation technologies. (a) Proposed photocatalytic degradation mechanism of pollutants using a g-C_3_N_4_/TiO_2_/CQD/EPVC membrane system, where synergistic integration of g-C_3_N_4_, TiO_2_, and CQDs enhances charge separation, spectral utilization, and self-cleaning behavior. Adapted with permission from ref. 138 copyright 2025, Elsevier. (b) Photoelectrocatalytic degradation mechanism and charge-transfer pathway in a dual Z-scheme Bi_2_MoO_6_/GQDs/TiO_2_ heterojunction for tetracycline degradation, where GQDs act as electron-transfer intermediates promoting directional charge migration and suppressing electron–hole recombination. Adapted with permission from ref. 153 copyright 2026, Royal Society of Chemistry.

Similarly, photoelectrocatalytic systems can further suppress charge recombination through externally applied bias, thereby enhancing ROS generation and mineralization efficiency.^219^ Li *et al.*^153^ developed a visible-light-driven Bi_2_MoO_6_/GQDs/TiO_2_ dual Z-scheme heterojunction for efficient photoelectrocatalytic tetracycline degradation. As illustrated in Fig. 16b, GQDs act as electron-transfer intermediates in the dual Z-scheme pathway, promoting directional charge migration and suppressing electron–hole recombination; ˙O_2_^−^ and h^+^ are the dominant reactive species leading ultimately to tetracycline degradation and mineralization.

Hybrid integration is particularly attractive because the practical efficiency and scalability of photocatalysis can be limited under complex wastewater conditions, while coupling with other techniques can improve pollutant removal, process sustainability, and operational efficiency.^220,221^ Process integration can therefore improve treatment robustness while reducing energy consumption and enhancing degradation. However, successful implementation requires careful optimization of operational compatibility, including pH, inorganic ions, residence time, and catalyst stability.^222,223^

### 7.4 Gap between laboratory studies and real environmental application

CDs/TiO_2_-based materials have shown high photocatalytic efficiencies for pollutant degradation,^108,185^ however the studies reviewed in this work have been conducted predominantly using synthetic pollutant solutions under controlled laboratory conditions. To date, studies evaluating their degradation performance in real wastewater matrices remain limited. This represents a major limitation for assessing their practical environmental applicability, because real wastewater contains dissolved organic matter, inorganic ions, suspended solids, and multiple coexisting contaminants that can interfere with light penetration, compete for adsorption sites, and scavenge reactive oxygen species.

Despite encouraging laboratory-scale demonstrations, only a limited number of studies have evaluated CDs/TiO_2_-based photocatalysts under environmentally relevant water matrices. Liu *et al.*^151^ studied the impact of different environmental water matrices on the photocatalytic activity of the CQD-pCN/TNs system. After 120 min of reaction, the system achieved TC degradation efficiencies of 86.2%, 80.0%, 60.0%, and 50.2% in ultrapure water, tap water, river water, and secondary effluent, respectively. The lower degradation efficiencies in secondary effluent and river water were mainly attributed to competing organic matter and inorganic ions, which can consume reactive species or compete with TC for adsorption sites. Hu *et al.*^224^ designed a dual-photoelectrode photoelectrocatalytic (PEC) cell consisting of a TiO_2_ nanoneedle array photoanode (TiO_2_ NNs/Ti mesh) and a nitrogen-doped carbon dot (NCD)-modified Co_3_O_4_ photocathode (NCDs/Co_3_O_4_/Ti mesh), which was designed to operate under low or no applied voltage. They also investigated the effects of real environmental matrices on the activity of the TiO_2_ NNs–NCDs/Co_3_O_4_ PEC system. After 120 min of reaction, the system achieved SDZ removal efficiencies of 97.66%, 96.31%, and 94.04% in lake water (LW), wastewater treatment plant effluent (WTPE), and pharmaceutical wastewater (PW), respectively. These results demonstrate that the dual-photoelectrode PEC system exhibits strong environmental adaptability and resistance to matrix effects, maintaining high SDZ removal efficiency in complex real-water matrices.

Long-term catalyst durability, fouling resistance, and photostability remain insufficiently explored. Consequently, pilot-scale validation and standardized performance-assessment protocols remain essential before practical environmental deployment can be achieved. Collectively, environmental remediation using CDs/TiO_2_ nanocomposites should be considered a multiscale optimization challenge involving catalyst design, reactor engineering, process integration, and environmental compatibility.

## 8 Stability, reusability, and environmental safety

For CDs/TiO_2_ nanocomposites to transition from laboratory-scale demonstrations to practical water-treatment technologies, long-term structural stability, sustained photocatalytic activity, and environmental safety must be rigorously validated. High degradation efficiency alone is insufficient if photocatalysts undergo progressive deactivation, release nanocarbon components into treated water, or generate transformation products with residual biological activity. Consequently, evaluation of catalyst durability and environmental compatibility has become a critical requirement for responsible large-scale implementation of photocatalytic remediation technologies. Representative reusability performance and toxicity assessment studies reported in the literature are summarized in Table 3.

**Table 3:** Stability, reusability, and toxicity assessment of representative CDs/TiO_2_ photocatalysts

Catalyst system	Retention of activity (%)	Toxicity assessment	Dominant deactivation mode	Ref.
Cu–TiO_2_/CQDs	94% after 6 cycles	Not evaluated	Blockage of surface pores of the catalyst by SMX and intermediates	141
CQDs/TiO_2_	67.89% after 5 cycles	Not evaluated	Loss of active catalyst material and deactivation of surface-active sites due to accumulation of intermediates; also, CQDs may bleach under prolonged UV exposure	140
Bi_2_WO_6_/C-dots/TiO_2_	92% after 3 cycles	Not evaluated	Minor loss of activity attributed to the leaching of Bi and Ti ions and loss of active sites during photocatalysis	121
T-CDs/TiO_2_	98% after 5 cycles	Not evaluated	Slight surface contamination or mass loss during catalysis	225
CDs-TLFO	∼89% after 6 cycles	Not evaluated	Some pollutant molecules are adhering to the surface of the catalyst, reducing available active sites	154
LJCQDs/TiO_2_	85% after 5 cycles	2 intermediates (TP1b, TP1c) showed slightly higher toxicity than TC	TC adsorption on the catalyst, minor CQD loss, and oxidation of Ti^3+^ to Ti^4+^	150
LPCQDs/TiO_2_				
RCDs/TiO_2_	84% after 6 cycles	Not evaluated	Loss of RCDs during the cycling	189
Sr-doped TiO_2_/CDs	63% after 4 cycles	Not evaluated	Material loss during recycling	161
CQD-pCN/TNs	78.3% after 5 cycles	Intermediates had no ecotoxicity except compound VIII (chronic toxicity to *Daphnia*)	Incomplete desorption of TC or/and intermediate	151
CQDs/g-C_3_N_4_/TiO_2_	57.9% after 3 cycles	Not evaluated	CQD detachment from the catalyst matrix	111
NPCDs/(001)TiO_2_/Ti_3_C_2_T_*x*_	86.55% after 5 cycles	Not evaluated	Loss of a small amount of photocatalyst	192
N-CQDs/TiO_2_	∼68% after 5 cycles	Not evaluated	Partial CQD leaching, catalyst loss during recovery, and the deactivation of surface-active sites due to byproduct accumulation	226
TiO_2_/CQDs	Good stability after 4 cycles	Final products showed negligible toxicity	Surface fouling by accumulated TC and intermediates	116
Co-NCDs/UTO	∼83% after cycle 6	Some TPs showed transiently higher toxicity than TCH; toxicity decreased with further degradation, though residual ecological hazards remained	Not evaluated	234

### 8.1 Photostability and cycling performance

Photostability refers to the ability of CDs/TiO_2_ nanocomposites to maintain photocatalytic activity and structural integrity during repeated irradiation cycles.^155,225^ Although TiO_2_ itself exhibits excellent chemical stability under irradiation conditions, incorporation of CDs introduces additional challenges, including gradual photobleaching and minor photocorrosion during prolonged operation.^140,226^

Continuous photocatalytic cycling may progressively modify CD surface chemistry because of persistent exposure to reactive oxygen species (ROS). Partial loss of CDs happens during long-term operation, especially in systems dominated by physical interaction rather than covalent Ti–O–C coupling.^171^ In addition, accumulation of partially oxidized intermediates on catalyst surfaces may block active sites and reduce photocatalytic efficiency even when crystal structure remains relatively unchanged.^116,227^

Cycling studies generally demonstrate moderate retention of photocatalytic activity over repeated reuse cycles, with only gradual reductions in photocatalytic efficiency.^154,189^ Post-reaction characterization using X-ray diffraction (XRD) often confirms preservation of the TiO_2_ crystalline framework, although attenuation of spectral intensities and minor surface-composition changes are frequently observed.^166,186,226^ These findings suggest that surface-level modifications rather than catastrophic structural collapse are primarily responsible for gradual activity loss.

Despite encouraging short-term cycling results, long-term continuous-flow assessments remain limited. Therefore, further investigation under environmentally relevant operational conditions is necessary to evaluate catalyst durability, fouling resistance, and interfacial stability during extended treatment processes.

### 8.2 Ecotoxicological assessment and safe-by-design considerations

Effective environmental remediation should ensure reduction of overall toxicity rather than merely achieving disappearance of the parent antibiotic compound.^228,229^ Incomplete mineralization during photocatalytic degradation may generate transient intermediates with altered bioavailability, residual antimicrobial activity, or elevated ecotoxicological effects relative to the original pollutant.^12,230^

Ecotoxicological assessment should therefore be integrated into broader safe-by-design strategies for photocatalytic material development. Bioassays involving microorganisms, algae, or aquatic invertebrates are commonly employed to compare toxicity before and after photocatalytic treatment.^231,232^ In addition to experimental bioassays, various computational approaches and predictive models, such as the Ecological Structure–Activity Relationships (ECOSAR) program, based on quantitative structure–activity relationship (QSAR) models, are widely used to assess the toxicity of parent antibiotics and their degradation intermediates.^233^

In many reported systems, photocatalytic degradation substantially decreases residual antimicrobial activity, indicating disruption of biologically active molecular structures. Nevertheless, partially oxidized intermediates formed during multistep degradation may transiently exhibit elevated toxicity before complete mineralization occurs.^151,165^ Xu *et al.*^116^ evaluated the environmental toxicity of tetracycline degradation products generated by the TiO_2_/CQDs photocatalytic system using bacterial toxicity tests. The toxicity of the treated solutions was investigated against *Escherichia coli* and *Staphylococcus aureus*. During photocatalytic degradation, the antibacterial activity of tetracycline gradually decreased, as indicated by the reduction and eventual disappearance of the inhibition zone against *S. aureus*. Furthermore, the treated solution after 30 min of photocatalysis showed negligible effects on *E. coli* growth compared with the blank control, indicating that the generated intermediates exhibited low toxicity. These results suggest that the TiO_2_/CQDs system not only degraded tetracycline but also reduced its biological toxicity, supporting its potential for environmentally safe wastewater treatment. Wang *et al.*^234^ developed a Co-NCDs/UTO photocatalytic system for degradation and evaluated the acute and chronic ecotoxicity of TCH and its degradation intermediates toward fish, daphnids, and green algae using the ECOSAR program based on the QSAR model. Some intermediates exhibited transiently higher toxicity than the parent TCH during the initial degradation stage, posing a potential accumulation risk. As degradation proceeded, the toxicity of the subsequent small-molecule intermediates generally declined, although certain ecological hazards remained. This finding highlights that pollutant degradation does not necessarily ensure complete detoxification and confirms the importance of toxicity assessment for evaluating the environmental safety of photocatalytic treatment systems. Therefore, evaluation of photocatalytic performance should include both parent-compound removal and systematic assessment of transformation-product toxicity.

Safe-by-design strategies emphasize strong interfacial bonding to minimize nanoparticle release, optimization of ROS pathways to promote deeper oxidation, and selection of environmentally benign precursors for CD synthesis. Collectively, catalyst durability, resistance to carbon release, and verified toxicity reduction represent interconnected requirements for environmentally responsible implementation of CDs/TiO_2_ photocatalysts. While short-term cycling studies indicate promising stability, long-term continuous-flow testing and comprehensive toxicological validation remain essential before large-scale deployment can be achieved.

## 9 Challenges and future perspectives

Despite substantial progress in the development of CDs/TiO_2_ nanocomposites for photocatalytic antibiotic degradation, large-scale environmental implementation remains constrained by unresolved challenges associated with interfacial electronic-structure optimization, mechanistic interpretation, standardized performance evaluation, long-term operational stability, and environmental safety validation. Although many reported systems demonstrate excellent laboratory-scale degradation efficiency, practical deployment requires coordinated advances in catalyst design, reactor engineering, environmental compatibility, and scalable solar-driven operation. The major future research directions and remaining technological challenges associated with CDs/TiO_2_ photocatalysts are summarized in Fig. 17.

**Fig. 17:**
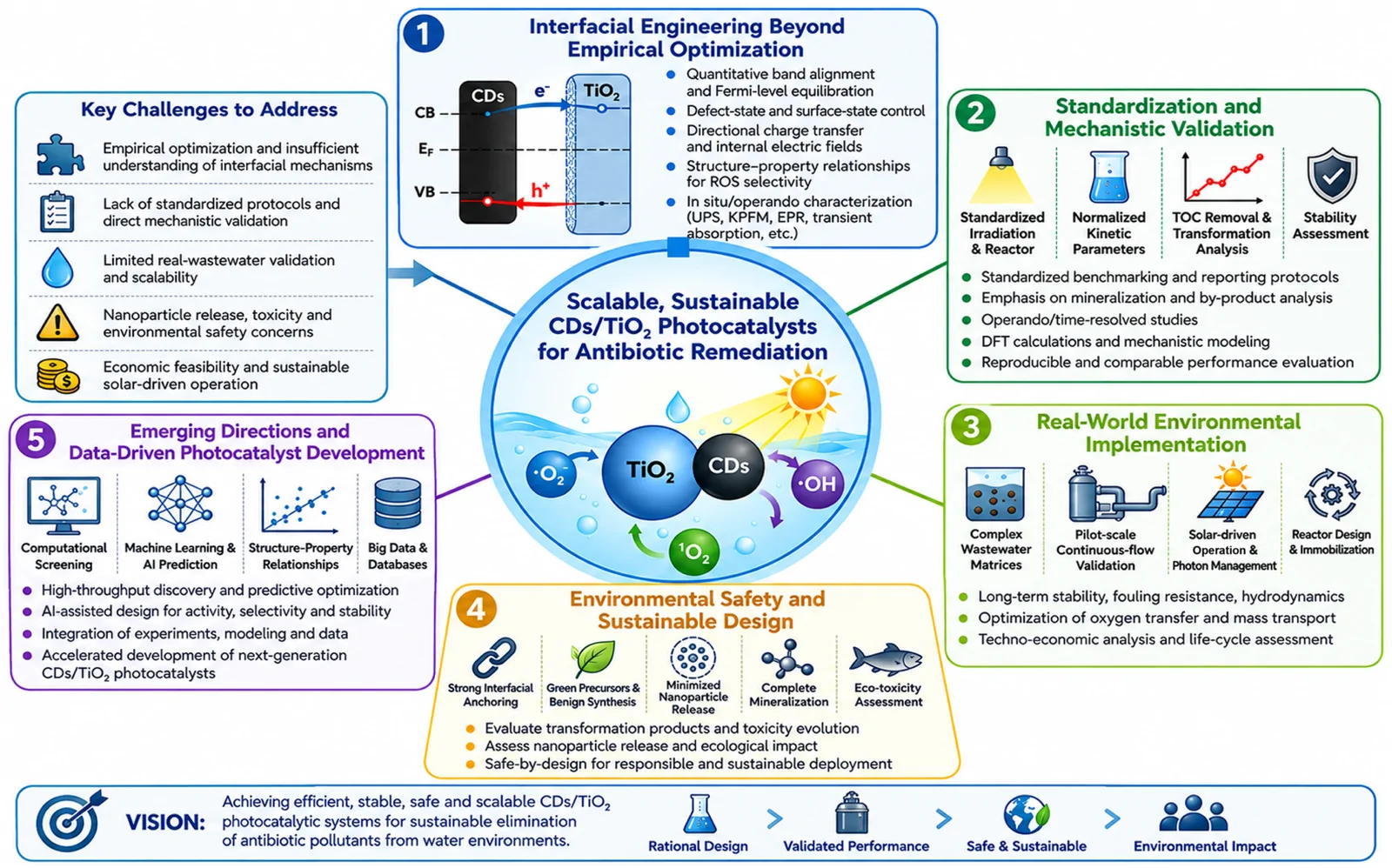
Major challenges and future research directions for CDs/TiO_2_ photocatalysts in environmental remediation. The framework highlights predictive interfacial engineering, mechanistic validation, standardized benchmarking, pilot-scale environmental implementation, environmental safety assessment, and AI-assisted photocatalyst design as key priorities for future development of sustainable antibiotic-remediation technologies.

### 9.1 Interfacial engineering beyond empirical optimization

Despite substantial advances in CDs/TiO_2_ photocatalyst development, optimization strategies remain largely empirical and are frequently based on trial-and-error adjustment of CD loading, precursor selection, and heteroatom doping composition rather than predictive electronic-structure design. Consequently, the relationships among interfacial electronic structure, defect-state distribution, charge-carrier dynamics, and ROS selectivity remain insufficiently understood.

Future investigations should prioritize quantitative determination of band-edge positions, Fermi-level equilibration, charge-transfer kinetics, and defect-state distributions using advanced *in situ* and *operando* spectroscopic techniques. Many studies still lack the use of complementary characterization techniques, including *in situ* X-ray photoelectron spectroscopy (XPS), ultraviolet photoelectron spectroscopy (UPS), and Kelvin probe force microscopy (KPFM). *In situ* XPS helps determine interfacial charge-transfer pathways, UPS provides information on work function and valence-band energetics, and KPFM maps surface-potential changes and charge accumulation, revealing built-in electric fields and charge-transfer directions. Together, these techniques can provide deeper insight into carrier migration pathways and interfacial electronic coupling under irradiation, helping to distinguish among different heterojunction mechanisms.

In addition, rational control of surface states and heteroatom incorporation remains challenging because excessive defect formation may simultaneously improve optical absorption while introducing additional recombination centers. Engineering spatially separated oxidation and reduction domains, internal electric fields, and directional carrier-migration pathways may provide effective strategies for simultaneously maximizing ROS generation efficiency and long-term stability. Establishing quantitative structure–property relationships linking CD surface chemistry, work-function alignment, and ROS selectivity with photocatalytic performance will therefore be essential for predictive catalyst design.

### 9.2 Standardization and mechanistic validation

Direct comparison among reported CDs/TiO_2_ photocatalytic systems remains difficult because experimental conditions vary substantially across studies. Differences in irradiation source, photon flux, catalyst dosage, reactor system, pollutant concentration, and analytical methodology frequently result in inconsistent degradation-rate comparisons and limited reproducibility.

Future studies should therefore prioritize development of standardized benchmarking protocols incorporating normalized kinetic parameters, photon-utilization efficiency, mineralization performance, and catalyst-stability assessment. In particular, greater emphasis should be placed on total organic carbon (TOC) removal and transformation-product analysis rather than parent-compound degradation alone.

Mechanistic interpretation also requires more rigorous experimental validation. Many proposed charge-transfer pathways are currently inferred indirectly from radical-scavenging experiments and photoluminescence behavior without complementary characterization techniques. Integration of *operando* spectroscopy, isotope-labeling strategies, density functional theory (DFT) calculations, and time-resolved spectroscopic analysis may provide more definitive mechanistic understanding of ROS-generation pathways and degradation selectivity.

### 9.3 Real-world environmental implementation

Although numerous CDs/TiO_2_ systems exhibit promising photocatalytic activity under laboratory conditions, validation under realistic environmental matrices remains limited. Most reported studies employ simplified aqueous systems containing single antibiotic compounds under controlled irradiation environments, whereas real wastewater contains complex mixtures of dissolved organic matter, inorganic ions, suspended solids, and coexisting contaminants that may strongly influence photocatalytic performance.

Future studies should therefore prioritize pilot-scale validation of CDs/TiO_2_ photocatalysts using authentic wastewater matrices, including hospital wastewater, pharmaceutical manufacturing effluents, agricultural wastewater, and aquaculture effluents, under continuous-flow operation. Long-term investigations should further evaluate catalyst stability, recovery and reusability, fouling resistance, hydrodynamic stability, treatment efficiency, energy demand, and photocatalytic performance under fluctuating environmental conditions.

In addition, large-scale implementation requires optimization of reactor architecture, photon-management strategies, oxygen-transfer efficiency, and catalyst immobilization techniques. Solar-driven photocatalytic systems are particularly attractive because they reduce dependence on external energy input and improve the sustainability of environmental remediation processes. However, efficient solar utilization under variable climatic conditions remains a major engineering challenge.

Economic feasibility additionally remains insufficiently explored. Future investigations should incorporate techno-economic analysis and life-cycle assessment to evaluate catalyst synthesis cost, operational energy consumption, catalyst recovery efficiency, and environmental impact associated with large-scale deployment.

### 9.4 Environmental safety and sustainable design

Long-term environmental compatibility remains one of the most important unresolved challenges associated with CDs/TiO_2_ photocatalysts. Although many studies report high degradation efficiency, systematic evaluation of nanoparticle release, transformation-product toxicity, and ecological impact remains relatively limited.

Future investigations should therefore integrate environmental safety assessment into photocatalyst design from the earliest stages of material development. Safe-by-design approaches emphasizing strong interfacial anchoring, environmentally benign precursors, minimized nanoparticle release, and enhanced mineralization capability may reduce long-term ecological risks.

Comprehensive ecotoxicological evaluation should include not only parent-compound removal but also toxicity evolution during multistep degradation, persistence of intermediate species, and potential impacts of released nanomaterials on aquatic ecosystems. Integration of microbial assays, aquatic toxicity studies, and long-term environmental monitoring will therefore be essential for responsible deployment of photocatalytic remediation technologies.

### 9.5 Emerging directions and data-driven photocatalyst development

Recent advances in computational modeling, machine learning, and artificial intelligence (AI)-assisted materials discovery offer new opportunities for accelerating photocatalyst development. Integration of high-throughput computational screening with experimental validation may enable predictive optimization of operating parameters, interfacial structure, band alignment, and ROS selectivity.

Machine-learning-assisted approaches may further support identification of structure–property relationships governing photocatalytic performance, catalyst stability, and degradation selectivity across structurally distinct antibiotic classes. Coupling experimental databases with computational modeling could significantly accelerate rational design of next-generation CDs/TiO_2_ photocatalysts with improved environmental compatibility and operational efficiency.

Future progress will therefore likely depend on interdisciplinary integration of photocatalysis, materials science, environmental engineering, computational chemistry, and data-driven materials design. Such collaborative strategies may accelerate development of scalable, environmentally responsible, and solar-responsive photocatalytic systems for sustainable antibiotic remediation.

## 10 Conclusions

This review highlights the critical role of heterointerface engineering in governing the photocatalytic performance of CDs/TiO_2_ nanocomposites for antibiotic degradation. The available literature demonstrates that interfacial Ti–O–C coupling, band alignment, defect-state modulation, and directional charge transfer collectively regulate visible-light utilization, reactive oxygen species (ROS) generation, adsorption behavior, degradation selectivity, and mineralization efficiency. Compared with pristine TiO_2_, incorporation of CDs generally improves charge separation, suppresses electron–hole recombination, and enhances photocatalytic degradation across structurally distinct antibiotic classes, including tetracyclines, fluoroquinolones, β-lactams, and sulfonamides. This review further demonstrates that photocatalytic degradation efficiency depends not only on catalyst composition, but also on the coupled interaction between interfacial electronic structure, ROS selectivity, environmental matrix effects, and antibiotic molecular properties. Although many CDs/TiO_2_ systems exhibit promising laboratory-scale performance, complete mineralization and toxicity elimination remain challenging because partially oxidized intermediates may persist during multistep degradation processes. Consequently, evaluation of photocatalytic performance should extend beyond parent-compound removal to include mechanistic validation, transformation-product analysis, long-term stability, and ecotoxicological assessment. Despite substantial progress in recent years, important challenges remain regarding standardized benchmarking, direct verification of charge-transfer pathways, real-wastewater validation, catalyst durability, nanoparticle-release assessment, and reactor-scale implementation. Addressing these limitations will require mechanistically guided interfacial engineering, *operando* characterization techniques, environmentally responsible catalyst design, and integrated optimization of solar-driven photocatalytic systems under realistic environmental conditions. Overall, bridging the gap between fundamental mechanistic understanding and practical environmental application will be essential for advancing CDs/TiO_2_ nanocomposites as sustainable technologies for mitigating antibiotic contamination and antimicrobial resistance in aquatic environments.

## Author contributions

Sewara J. Mohammed: writing – original draft, writing – review & editing, project administration, supervision, validation. Eman O. Hamarashid: writing – original draft, writing – review & editing, resources, software. Hastyar H. Najmuldeen: writing – review & editing, visualization. Dana A. Kader: writing – review & editing, investigation. Mohammed K. Sidiq: writing – review & editing, validation. Faiq H. S. Hussain: writing – review & editing, visualization. Awat S. Mohammed: writing – review & editing, resources. Fayez Alghofaili: writing – review & editing, software, funding acquisition. Govar H. Hamasalih: writing – review & editing, visualization. Shakhawan A. Mhamad: writing – review & editing, investigation. Yar M. Faqi-Khedr: writing – review & editing, resources. Kawan F. Kayani: writing – review & editing, software. Othman K. Hamaamin: writing – review & editing, investigation. Shujahadeen B. Aziz: writing – review & editing, project administration, validation. All authors reviewed the manuscript.

## Conflicts of interest

There are no conflicts to declare.

## Data Availability

No data was used for the research described in the article.
